# Incidence of Lyme Borreliosis in Finland: Exploring Observed Trends Over Time Using Public Surveillance Data, 2015–2020

**DOI:** 10.1089/vbz.2022.0047

**Published:** 2023-04-12

**Authors:** Jozica Skufca, Nick De Smedt, Andreas Pilz, Andrew Vyse, Elizabeth Begier, Maxim Blum, Margarita Riera, Bradford D. Gessner, James H. Stark

**Affiliations:** ^1^ Epidemiology and Pharmacovigilance, P95, Koning Leopold III Iaan 1, Leuven, Belgium.; ^2^ Vaccines, Pfizer Corporation Austria, Wien, Vienna, Austria.; ^3^ Vaccines Medical Affairs, Pfizer Ltd, Walton Oaks, Tadworth, United Kingdom.; ^4^ Vaccine Clinical Research and Development, Pfizer Inc, Pearl River, New York, USA.; ^5^ Vaccines Medical Development and Scientific/Clinical Affairs, Pfizer Inc, Collegeville, Pennsylvania, USA.

**Keywords:** Lyme borreliosis, tick-borne disease, surveillance, spatial epidemiology, incidence, Finland

## Abstract

**Background:**

Lyme borreliosis (LB) is a tick-borne zoonotic disease endemic in many European countries, including Finland. We describe the incidence, time trends, and geographical distribution of LB in Finland for the period 2015–2020. The data generated can help inform public health policy, including prevention strategies.

**Methods:**

We retrieved online-available LB cases and incidence from two Finnish national databases. Microbiologically confirmed LB cases were identified from the National Infectious Disease Register and clinically diagnosed LB cases from the National Register of Primary Health Care Visits (Avohilmo), with the total LB cases equal to the sum from these two sources.

**Results:**

A total of 33,185 LB cases were reported for the 2015–2020 period, of which 12,590 (38%) were microbiologically confirmed and 20,595 (62%) were clinically diagnosed. The average annual national incidences for total, microbiologically confirmed, and clinically diagnosed LB were, respectively, 99.6, 38.1, and 61.4 per 100,000 population. The total LB incidence was highest in the south to southwestern coastal areas by the Baltic Sea and in eastern areas, with average annual incidences of 109.0 to 207.3/100,000. The Åland Islands were a hyperendemic region with an average annual incidence of 2473.9/100,000. The highest incidence was among persons aged >60 years, peaking at age 70–74 years. Most cases were reported between May and October, with a peak in July and August.

**Conclusions:**

The incidence of LB varied substantially by hospital district, and many regions reached incidences comparable with other high incidence countries, suggesting preventive measures such as vaccines may be an efficient use of resources.

## Introduction

Lyme borreliosis (LB) is an infectious vector-borne disease caused by the spirochete *Borrelia burgdorferi* sensu lato (s.l.) and transmitted through the bite of ticks of genus *Ixodes*, that results in multiple clinical manifestations, which include erythema migrans (EM) and more severe disseminated manifestations, such as Lyme neuroborreliosis (LNB) and Lyme arthritis (LA) (Cardenas-de la Garza et al, 2019).

LB surveillance exists in many European countries, and since 2019, LNB is mandatorily notifiable in EU (Hy and Muhhamad, 2018). However, not all countries have mandatory reporting of LB, and the type of surveillance, reporting and LB case definitions vary widely (Lorenc et al, 2017). Although the incidence rates of LB across Europe are influenced by geographical, environmental, and climatic factors, as well as human behaviors, including recreational activity, the heterogeneity found among surveillance systems within Europe further complicates the comparison of the incidence between countries (van den Wijngaard et al, 2017). Under reporting and over reporting, as well as differences in case definitions, diagnostic difficulties, and different laboratory methods, are recognized issues for LB diagnosis and surveillance.

Furthermore, data collection may differ (*e.g.*, epidemiological surveys vs. laboratory-based notification systems), and data collection may not be representative of the whole country (*e.g.*, only including high-incidence regions). Accordingly, highly divergent incidence rates for LB have been reported between and within some countries (van den Wijngaard et al, 2017). In Finland, LB cases are identified either through routine mandatory laboratory-based surveillance and reported to the National Infectious Disease Register (NIDR) or through a clinical diagnosis of LB reported to the National Register of Primary Health Care Visits (Avohilmo) (Feuth et al, 2020).

Routine mandatory laboratory-based surveillance includes LB treated in hospitals, outpatient clinics of hospitals, primary health care, private general practitioners (GPs), and occupational health. Since 1995, LB-positive cases have been identified by routine laboratory testing of *Borrelia*-specific immunoglobulin G or immunoglobulin M antibodies in serum or cerebrospinal fluid. Serology is based on two-tier testing whereby *Borrelia*-specific antibodies are detected using a sensitive enzyme immunoassay followed by a more specific immunoblot. At the time, eight laboratories (both public and private units) in Finland perform LB laboratory diagnostics; LB-positive findings are reported directly from the microbiological laboratory's electronic information system to the NIDR (Sajanti et al, 2017). Cases in the NIDR are considered “microbiologically confirmed LB cases” and represent mostly disseminated LB (Feuth et al, 2020; Finnish Institute for Health and Welfare [THL]; Sajanti et al, 2017).

Patients presenting at GPs in public outpatient primary health care centers across Finland are clinically diagnosed without laboratory testing. When a case is clinically diagnosed, a GP enters the International Classification of Diseases, Tenth Revision (ICD-10) code for LB (A69.2) in the patient records. Beginning in 2011, these cases have been automatically reported to Avohilmo. Cases in Avohilmo are considered “clinically diagnosed” and mostly represent EM. From 2018 onward, in addition to the laboratory surveillance of microbiologically confirmed disseminated LB cases recorded in NIDR, Avohilmo was introduced into the routine surveillance of LB in Finland (Feuth et al, 2020; Finnish Institute for Health and Welfare [THL]; Sajanti et al, 2017).

The burden of LB in Finland has been investigated previously using different methods, in different periods, and among different populations. A study using historical serum samples from 1968 to 1972 among the general population in Finland reported a seroprevalence of 20.0% (Cuellar et al, 2020), at a time when the economy was heavily based on agriculture and forestry, with one-third of the study participants working in these sectors. More recently, LB seroprevalence among the adult population in Finland in 2011 was estimated at 3.9% (van Beek et al, 2018). A study analyzing both Avohilmo and NIDR data reported an annual incidence of microbiologically confirmed disseminated LB that increased from 7/100,000 population in 1995 to 31/100,000 population in 2014 and of clinically diagnosed LB cases (EM) from 44/100,000 population in 2011 to 61/100,000 population in 2014 (Sajanti et al, 2017).

This study explored the spatial distribution and trends in the incidence of microbiologically confirmed LB and clinically diagnosed LB for the 2015–2020 period in Finland to assess incidence and identify high-risk groups and areas that could benefit from preventive measures, including vaccines. The data generated provide updated information that can inform public health policy.

## Materials and Methods

### Data sources and variables

To assess all reported LB cases in Finland (clinically diagnosed EM and microbiologically confirmed disseminated LB), we retrieved online-available data from Avohilmo and NIDR, with both systems maintained by THL. Both registers contain data from the entire country and by hospital districts (HDs) and municipalities. The Finnish national health care system is organized into 20 geographically and administratively defined HDs with between 6 and 35 municipalities, which are responsible for primary and specialized care. The autonomous region of the Åland Islands is considered the 21st HD.

Cases reported to Avohilmo and NIDR are mostly mutually exclusive since cases of EM are usually diagnosed only clinically and not laboratory confirmed in the primary health care setting (Avohilmo), whereas cases in NIDR must be microbiologically confirmed. Exceptions could occur if GPs order laboratory testing for EM despite recommendations not to do so, or if they test for disseminated LB at the primary health care level. A database linkage study showed that the overlap between the registers was 6.3% in 2014 (Feuth et al, 2020).

Data on microbiologically confirmed LB reported from laboratories to the NIDR were obtained online. The register provided information about number of cases and incidence (cases per 100,000 population) by HDs, municipalities, age groups, sex, year, and month (Finnish Institute for Health and Welfare [THL]). The location of reported cases reflected the place of residence or diagnosis and not the place where the exposure occurred.

Data on clinically diagnosed LB collected by GPs in primary health care units and recorded in Avohilmo were obtained from an online platform (Finnish Institute for Health and Welfare [THL]), including the number of cases by HDs, municipalities, age groups, year, and month. Data by sex were not available.

### Statistical methods

Data on LB were extracted from the two online platforms on January 29, 2021, for the period from January 1, 2015, to December 31, 2020. Relevant population denominators were derived from NIDR data where incidence and microbiologically confirmed case counts were presented to allow the use of the same population denominator to estimate microbiologically and clinically diagnosed incidences and total LB incidence. Clinically diagnosed LB incidence was calculated by dividing the number of cases reported in Avohilmo by the population size.

Total LB incidence was estimated as the sum of cases in NIDR (microbiologically confirmed) and Avohilmo (clinically diagnosed) divided by the population size. Although there is a certain degree of overlap between the two databases (6.3%; Feuth et al, 2020), we chose to simplify the analysis and provide an estimate of the total LB incidence without adjusting for the overlap, given the minimal impact. Because NIDR did not report municipality-level incidence data, population data for these analyses were obtained directly from the Statistics Finland statistical database (2021).

The 95% confidence intervals (95% CIs) for the incidence were calculated using the exact binomial method (Wilson, 1927). The average annual number of cases and the average annual incidence for the 6-year period (2015–2020) were calculated as the weighted averages of the annual number of cases and the annual incidence estimates, respectively, with the weights equal to the annual denominators. The 95% CI for the average incidence was calculated using the combined stratum-specific F-distribution CIs (Waller et al, 1994). After conducting the primary analysis, the incidence from the Åland Islands was substantially greater than that of all the other HDs. Therefore, we calculated the average annual cases and incidence of total LB, including only the mainland of Finland (excluding Åland). All analyses were performed using the statistical software R, version 4.0.4. (R Core Team, 2016).

Because only publicly available data were utilized in this study, the requirement for Institutional Review Board approval was waived.

## Results

### Time trends

Between 2015 and 2020, the total number of LB cases (clinically diagnosed and microbiologically confirmed) in Finland ranged from 4917 to 6006 per year. A total of 33,185 LB cases were reported for the 6-year period, with the annual national incidence ranging from 90.0 (95% CI: 87.5–92.5) to 108.8 (95% CI: 106.1–111.6) per 100,000 population.

The number of clinically diagnosed LB cases from Avohilmo ranged from 2984 to 3777 per year. A total of 20,595 clinically diagnosed LB cases were reported for the whole period, equaling 62% of the total LB cases (range by HDs: 31–88%). The annual incidence of clinically diagnosed cases ranged from 54.6 (95% CI: 52.7–56.6) to 68.5 (95% CI: 66.4–70.7) per 100,000 population.

**FIG. 1.**
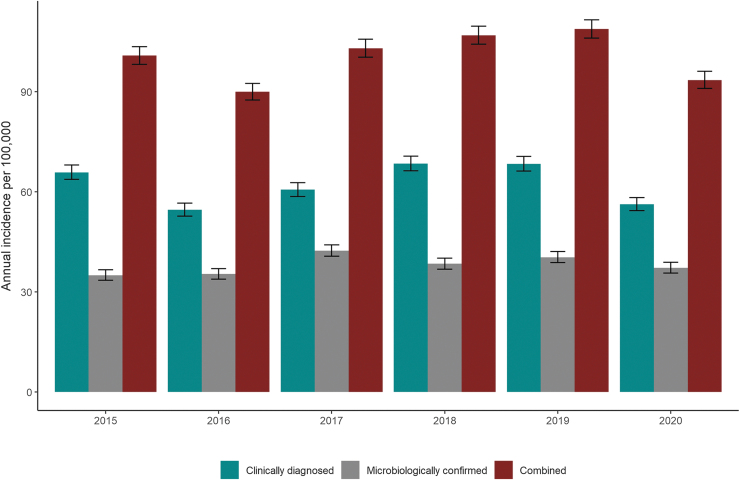
Annual incidence (per 100,000 population, ±95% CI) of clinically diagnosed LB cases reported in the Register for Primary Health Care Visits (Avohilmo), microbiologically confirmed LB cases reported in the NIDR, and both combined, Finland 2015–2020. CI, confidence interval; LB, Lyme borreliosis; NIDR, National Infectious Disease Register.

The number of microbiologically confirmed LB cases from NIDR ranged from 1913 to 2331 per year. A total of 12,590 microbiologically confirmed LB cases were reported for the whole period, equaling 38% of the total LB cases (range by HDs: 12–69%). In contrast to all other HDs, only Åland reported more microbiologically confirmed (69%) than clinically diagnosed (31%) LB cases. The annual incidence for microbiologically confirmed cases in Finland ranged from 35.0 (95% CI: 33.5–36.6) to 42.3 (95% CI: 40.7–44.1) per 100,000 population (Fig. 1).

### Geographic distribution

The average annual incidence of the whole country (average of all HDs) for the years 2015–2020 was 99.6/100,000 population for total, 61.4/100,000 population for clinically diagnosed and 38.1/100,000 population for microbiologically confirmed LB. Excluding the hyperendemic island of Åland, the average annual incidences of total, clinically diagnosed, and microbiologically confirmed LB for the 20 HDs in the mainland of Finland were 86.9/100,000 population, 57.7/100,000 population, and 29.1/100,000 population, respectively.

**FIG. 2.**
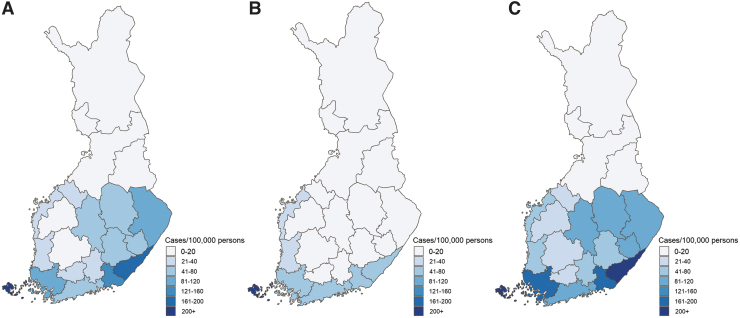
Average annual incidence (per 100,000 population) by 21 Finnish HDs for the years 2015–2020. **(A)** Clinically diagnosed LB. **(B)** Microbiologically confirmed LB. **(C)** Combined clinically diagnosed and microbiologically confirmed LB. HDs, hospital districts.

**FIG. 3.**
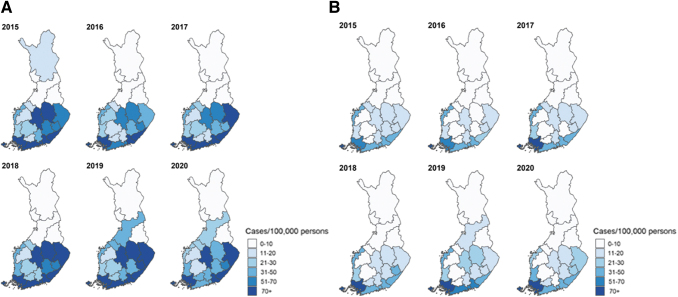
Annual incidence (per 100,000 population) by 21 Finnish hospital districts during 2015–2020. **(A)** Clinically diagnosed LB. **(B)** Microbiologically confirmed LB.

The average annual total LB incidence varied almost 300-fold by HD (Table 1), with a concentration of higher incidence in the south and southeastern HDs (Fig. 2). We report detailed data by years, HDs, and municipalities in Supplementary Appendix Tables SA1 to SA3. For the 2015–2020 period, the incidence was stable by HDs without a clear trend in reported clinically diagnosed or microbiologically confirmed LB. A slight increase was observed in clinically diagnosed cases in the Kymenlaakso and Pohjois-Pohjanmaa HDs in 2019–2020 (Fig. 3; Supplementary Appendix Tables SA1 and SA2). Incidence was also stable by municipalities, with the highest incidences reported in 16 municipalities of the Åland HD. Most LB cases for the years 2015–2020 were reported in urban municipalities/main cities (Supplementary Appendix Table SA3).

**Table 1.** Average Annual Cases and Incidence (Per 100,000 Population, ±95% CI) of Microbiologically Confirmed Lyme Borreliosis Cases Reported in the National Infectious Diseases Register, of Clinically Diagnosed Lyme Borreliosis Cases Reported in the Register for Primary Health Care Visits (Avohilmo), and of Both Combined by 21 Finnish Hospital Districts for the Years 2015–2020

No.	Hospital district	Population	Clinically diagnosed LB (Avohilmo)		Microbiologically confirmed LB (NIDR)		Total LB (Avohilmo + NIDR)	
			Cases	Incidence [95% CI]	Cases	Incidence [95% CI]	Cases	Incidence [95% CI]
1	The Åland Islands	29,405	222	756.8 [661.3–862.2]	504	1717.1 [1572.8–1871.1]	726	2473.9 [2300.4–2657.0]
2	Etelä-Karjala	130,030	216	165.6 [144.3–189.2]	54	41.7 [31.3–54.3]	270	207.3 [183.4–233.5]
3	Kymenlaakso	169,398	216	127.8 [111.4–146.0]	82	48.4 [38.5–60.1]	298	176.3 [156.9–197.4]
4	Varsinais-Suome	479,456	467	97.4 [88.8–106.7]	364	75.9 [68.3–84.1]	831	173.3 [161.8–185.5]
5	Helsingin ja Uudenmaa	1,645,715	1202	73.2 [69.1–77.4]	723	43.8 [40.7–47.2]	1925	117.0 [111.8–122.3]
6	Pohjois-Karjala	166,954	152	91.2 [77.8–106.5]	30	17.8 [12–25.4]	182	109.0 [94.1–125.7]
7	Keski-Suome	252,398	190	75.3 [65.1–86.8]	39	15.2 [10.8–20.9]	229	90.6 [79.3–103.1]
8	Pohjois-Savo	245,495	179	72.8 [62.7–84.1]	40	16.3 [11.7–22.1]	219	89.1 [77.9–101.6]
9	Itä-Savo	42,407	27	64.1 [42.4–93.1]	9	20.5 [9.3–39.2]	36	84.6 [59.3–117.1]
10	Vaasa	169,883	57	33.6 [25.5–43.5]	64	37.8 [29.2–48.2]	121	71.4 [59.4–85.2]
11	Etelä-Savo	101,785	48	47.0 [34.8–62.2]	11	11.1 [5.8–19.6]	59	58.1 [44.4–74.8]
12	Satakunna	219,512	62	28.2 [21.7–36.2]	52	23.9 [18–31.3]	114	52.2 [43.1–62.6]
13	Päijät-Hämee	209,313	62	29.6 [22.8–37.9]	28	13.4 [8.9–19.3]	90	43.0 [34.6–52.9]
14	Keski-Pohjanmaa	77,661	21	27.0 [17.0–41.1]	8	10.1 [4.4–19.9]	29	37.1 [25.0–53.3]
15	Kanta-Hämee	173,245	38	21.9 [15.6–30.0]	17	9.7 [5.7–15.6]	55	31.6 [23.9–41.1]
16	Pirkanmaa	531,303	110	20.7 [17.1–25.0]	35	6.6 [4.7–9.2]	146	27.4 [23.1–32.2]
17	Etelä-Pohjanmaa	194,895	37	19.2 [13.5–26.3]	5	2.6 [0.9–5.9]	42	21.7 [15.7–29.3]
18	Pohjois-Pohjanmaa	408,905	55	13.5 [10.5–17.3]	24	5.9 [3.9–8.8]	80	19.4 [15.6–24.0]
19	Lapi	117,615	10	8.8 [4.3–16.0]	4	3.7 [1.2–9.0]	15	12.5 [7.0–20.6]
20	Länsi-Pohja	62,108	4	6.2 [1.8–16.0]	3	5.4 [1.3–14.9]	7	11.5 [4.8–23.5]
21	Kainuu	74,295	4	5.2 [1.6–13.3]	2	3.1 [0.6–10.3]	6	8.3 [3.3–17.7]
	**Total Finland**	**5,501,777**	**3379**	**61.4 [59.4–63.5]**	**2099**	**38.1 [36.5–39.8]**	**5478**	**99.6 [97.0–102.2]**

*Note:* Numbered HDs (#) correspond to the numbered areas in Fig. 2C.

CI, confidence interval; HDs, hospital districts; LB, Lyme borreliosis; NIDR, National Infectious Disease Register.

### Demographic characteristics

**FIG. 4.**
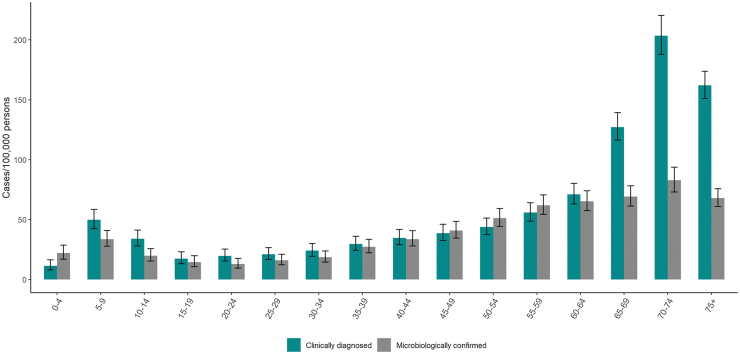
Average annual incidence (per 100,000 population, ±95% CI) of clinically diagnosed LB cases reported in the Register for Primary Health Care Visits (Avohilmo) and of microbiologically confirmed LB cases reported in the NIDR by age groups in Finland in the 2015–2020 period.

Among persons <40 years, clinically diagnosed and microbiologically confirmed LB cases peaked among children aged 5–9 years. However, the highest incidences were observed among persons >60 years, peaking at age 70–74 years. The incidence of clinically diagnosed LB was higher than microbiologically confirmed LB in all age groups, except in the age groups 0–4 and 45–59 years (Fig. 4).

We did not have information by sex for clinically diagnosed LB cases, only for microbiologically confirmed LB cases. Of all microbiologically confirmed LB cases, 50.3% (6328/12,590) occurred in females and 49.7% in males (6262/12,590). We did not observe any significant sex-specific differences in incidences across age groups, except a slightly, but significantly, higher incidence in males 75 years and older, with average annual incidences per 100,000 population of 85.0 (95% CI: 72.6–99.4) in males versus 62.2 (95% CI: 53.8–72.0) in females (data not shown).

### Seasonality

**FIG. 5.**
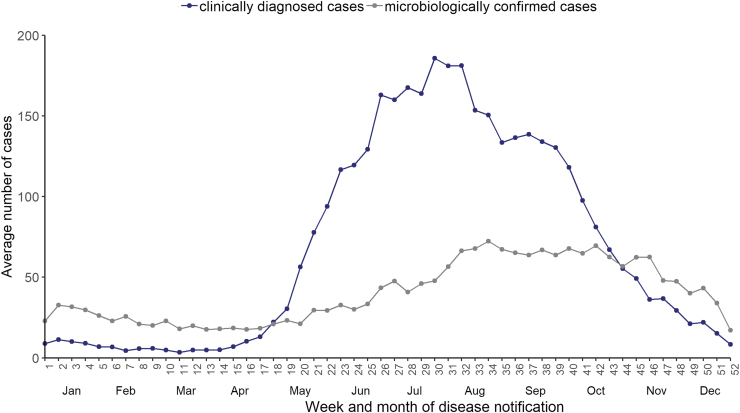
Average number of weekly clinically diagnosed cases reported in the Register for Primary Health Care Visits (Avohilmo) and of weekly microbiologically confirmed LB cases reported in the NIDR in Finland in the 2015–2020 period.

During the 2015–2020 period, 82% of the cases were reported between end-May (week 22) and mid-October (week 41), peaking at the end of July and August (weeks 30–32), with an average of 179 cases per week. Half of all microbiologically confirmed cases (51%) were reported between August (week 31) and November (week 48) (Fig. 5).

## Discussion

By using the online-available data from two nationwide registers, we examined the changes in the incidence and geographic distribution of clinically diagnosed LB (reflecting EM) and of microbiologically confirmed LB (reflecting disseminated forms) in Finland during 2015–2020. Approximately 3379 cases of EM and 2099 cases of disseminated LB were reported annually, resulting in an average annual incidence of 99.6/100,000 population (5478 cases).

Our study estimated higher LB incidence compared with data from 1995 to 2014, although the incidence seems to have reached a plateau in the period 2015–2020, following the increase observed in the previous period (Sajanti et al, 2017). The incidence in Finland is among the highest in Europe, comparable with Lithuania (Petrulioniene et al, 2020), Austria, and Slovenia (Smith and Takkinen, 2006). In some Finnish regions, the incidence is as high as that in the northeastern United States (Schwartz et al, 2017). Note, comparing incidence of LB across countries is always limited by the heterogeneity in surveillance systems and diagnostic protocols.

Our spatially granular data enabled an analysis by HDs and municipalities. This showed a substantial geographic variation of LB in Finland, with annual incidences varying from 8.3/100,000 in Kainuu to 207.3/100,000 in Etela-Karjala, and an exceptionally high incidence in Åland, at 2473.9/100,000. Differences in LB incidence between HDs could potentially be affected by a variety of factors, including geographic characteristics influencing tick populations, prevalence, and distribution of tick-borne pathogens, or human factors, such as outdoor activity (Laaksonen et al, 2018). In total, six HDs had an annual incidence of more than 90/100,000, which raises the question of whether these substantial regional variations support regionally focused approaches to address LB or whether a widespread national prevention program would be justified.

In addition, most areas with high LB incidence corresponded to high population density areas, indicating that most of the Finnish population live in potential high-incidence areas, which may increase the efficiency of a national approach. In the United States, a region with >10 confirmed cases/100,000 population is considered high incidence, and most regions of Finland would be classified as such (Kugeler et al, 2015). These high-incidence areas also have a high abundance of *Ixodes ricinus* ticks infected with *B. burgdorferi* s.l. (Laaksonen et al, 2017, Sormunen et al, 2020). Prevalence of *B. burgdorferi* s.l. in ticks can vary greatly within the country, from ∼14% in southern Finland (Laaksonen et al, 2017), 23% in southwestern Finland (Sormunen et al, 2016), to up to 55% in Helsinki (Junttila et al, 1999), although the latter prevalence may be due to a sampling bias.

We observed a bimodal age-specific distribution of clinically diagnosed and microbiologically confirmed LB cases. A similar age distribution has been noted in a previous Finnish study (Sajanti et al, 2017) and in other studies of LB in Europe (Bennet et al, 2006, 2007, Eliassen et al, 2017, Enkelmann et al, 2018). One explanation for the obvious peak in age groups >60 years may be that outdoor activities in Finland, such as mushroom and berry picking or gardening, are popular; older Finns may be more accustomed to these activities and are thus more exposed to infected ticks (Sajanti et al, 2017). A recent seroprevalence study in Finland demonstrated an increasing seroprevalence by age (van Beek et al, 2018). We also observed a higher incidence of clinically confirmed LB cases versus microbiologically confirmed LB cases in the 65+ years age group. This may reflect a higher incidence of EM, which does not require laboratory confirmation among this age group.

Reported LB clinical cases peaked at the end of July, whereas the LB laboratory confirmed cases presented a time lag with the clinical reporting, confirming the observations of Sajanti et al (2017).

The strength of our study is the comprehensive assessment of reported LB cases in Finland by using the Avohilmo and NIDR databases (Feuth et al, 2020). Many European countries do not have mandatory reporting of LB, or only have laboratory-based LB surveillance (Lorenc et al, 2017). However, without clinically diagnosed EM, the overall LB burden is greatly underestimated. The results of this study represent the burden of notified LB in Finland, including both mandatory reported microbiologically confirmed LB, representing disseminated forms of LB, and reported cases of clinically diagnosed LB, representing EM.

Moreover, data available in these databases include the general population from the whole country and reported by all GPs involved in the diagnosis of LB in Finland, although in Avohilmo, occupational and private health care are not included. Also, after the GPs enter an ICD-10 code for LB, each case is notified to Avohilmo automatically from GP systems (and Avohilmo is updated weekly) without the need for active reporting (Feuth et al, 2020). This helps reduce underreporting and provides timely data. Both registers also provided the most recent data up to the end of 2020, as well as detailed geographic data stratified on a subcountry level by HDs and municipalities, allowing the identification of high-incidence areas at a very granular level.

Our study has some limitations. First, this analysis combined incidence data from both databases. A previous study suggests that ∼6.3% of cases are found in both databases; thus, the estimates for the overall number of LB cases provided in this study are slightly higher than the absolute number of reported cases. Second, the number of clinically diagnosed LB cases could have been underestimated since Avohilmo does not include occupational or private health care visits, and in 2014 it was estimated that ∼13% of persons with microbiologically confirmed LB utilized these sources (Feuth et al, 2020, Keskimaki et al, 2019).

Also, compared with other European countries, a relatively low percentage (62%) of all cases were clinically diagnosed and thus presumed EM versus microbiologically confirmed and thus presumed disseminated LB (38%); this may indicate underreporting or diagnosis of EM by GPs, or failure of patients with EM to present for care such that it progresses to disseminated LB. For example, German surveillance data indicate a much higher percentage of clinically diagnosed EM (95%) versus disseminated LB (5%) (LA, LNB) (Enkelmann et al, 2018). Third, available data in the NIDR did not allow us to estimate disseminated LB cases by manifestation (*e.g.*, LNB and LA).

Furthermore, individuals may have received a laboratory test for an EM case despite standard of care practices, resulting in higher perceived LB cases than truly exist. The poor sensitivity of laboratory data for EM cases (∼50%) supports the lack of use of laboratory confirmation of an EM case, and thereby emphasizing the clinical diagnosis for EM (Steere et al, 2016). Sensitivity and specificity of disseminated outcomes, however, is greatly improved with traditional two-tier laboratory diagnostic testing (Lantos et al, 2020, Steere et al, 2016).

Next, like in other LB surveillance systems, information about the place of infection was not available, and the location where the infection occurred might be different than the place of residence, for example, people could get infected while spending time at their summer cottages but could get diagnosed in another area. The value of the spatial analysis could be increased by including the place of infection in the reporting, although this would come with a substantial workload increase, and may not be cost-effective in countries with high incidence. Finally, the data presented in this study illustrate the incidence of disease, which is one measure to quantify burden; however, measurement of disability-adjusted life years as quantified by van den Wijngaard et al (2015) can illuminate which aspects of disease provide the greatest disability.

## Conclusions

The high LB incidence and hyperendemicity in some areas confirmed in this study suggest that LB remains an important health problem in Finland. The most affected populations are children and older persons. By including both early EM and disseminated LB forms, our data add to the comprehensive understanding of the overall burden of LB in Finland. Moreover, by analyzing and reporting spatially granular data, we identified areas with highest incidence to increase public awareness of areas with highest exposure risks. More studies are needed to increase our understanding of risk factors for LB among different age groups to better target preventive measures. Our study will support public health decision-making for investment in preventive measures.

## Supplementary Material

Supplemental data

Supplemental data

Supplemental data

## Data Availability

All data generated or analyzed during this study are included in this published article.

## Supplementary Material

Supplementary Appendix Table SA1

Supplementary Appendix Table SA2

Supplementary Appendix Table SA3
