# Supplementary material for: Incidence of Lyme Borreliosis in Finland: Exploring Observed Trends Over Time Using Public Surveillance Data, 2015–2020
Source: Vector Borne Zoonotic Dis. 2023 Apr 12;23(4):256–64. doi: 10.1089/vbz.2022.0047 (PMC10122252; doi:10.1089/vbz.2022.0047)
Supplement: Supplemental data [file Suppl_AppendixTableS1.docx]

**Table 1.** Number of cases (N) and incidence (per 100,000 residents, ± 95% CI) of clinically diagnosed Lyme borreliosis reported in the Register for Primary Health Care Visits (Avohilmo) by year and Finnish hospital districts (HD), 2015–2020.

|  | **2015** | | **2016** | | **2017** | | **2018** | | **2019** | | **2020** | |
| --- | --- | --- | --- | --- | --- | --- | --- | --- | --- | --- | --- | --- |
| **HD** | **N** | **Incidence [95% CI]** | **N** | **Incidence [95% CI]** | **N** | **Incidence [95% CI]** | **N** | **Incidence [95% CI]** | **N** | **Incidence [95% CI]** | **N** | **Incidence [95% CI]** |
| **Ahvenanmaa** | 305 | 1,054.78 [943.36; 1,179.20] | 205 | 706.97 [616.85; 810.15] | 244 | 834.64 [736.62; 945.58] | 205 | 695.18 [606.56; 796.64] | 199 | 668.03 [581.68; 767.11] | 177 | 590.51 [509.88; 683.81] |
| **Etelä-Karjalan** | 280 | 212.51 [189.05; 238.86] | 218 | 166.15 [145.52; 189.70] | 176 | 134.80 [116.32; 156.23] | 229 | 176.33 [154.94; 200.67] | 157 | 121.95 [104.31; 142.55] | 232 | 181.32 [159.45; 206.17] |
| **Etelä-Pohjanmaan** | 33 | 16.63 [ 11.84;  23.36] | 55 | 28.97 [ 22.26;  37.70] | 30 | 15.22 [ 10.67;  21.73] | 40 | 20.40 [ 14.98;  27.78] | 33 | 16.99 [ 12.10;  23.87] | 33 | 17.05 [ 12.14;  23.94] |
| **Etelä-Savon** | 73 | 70.28 [ 55.90;  88.35] | 36 | 34.83 [ 25.16;  48.21] | 33 | 32.17 [ 22.91;  45.18] | 54 | 53.21 [ 40.79;  69.41] | 49 | 48.88 [ 36.98;  64.61] | 42 | 42.42 [ 31.39;  57.33] |
| **Helsingin ja Uudenmaan** | 1,259 | 78.72 [ 74.49;  83.19] | 1,126 | 69.55 [ 65.61;  73.73] | 1,295 | 79.07 [ 74.88;  83.49] | 1,443 | 87.37 [ 82.98;  92.00] | 1,248 | 74.85 [ 70.81;  79.12] | 850 | 50.12 [ 46.87;  53.61] |
| **Itä-Savon** | 30 | 68.10 [ 47.71;  97.20] | 25 | 57.50 [ 38.95;  84.87] | 19 | 44.15 [ 28.27;  68.95] | 28 | 66.32 [ 45.89;  95.84] | 33 | 80.38 [ 57.24; 112.86] | 28 | 69.38 [ 48.01; 100.26] |
| **Kainuun** | 3 | 3.94 [ 1.34;  11.59] | 6 | 7.96 [ 3.65;  17.37] | 1 | 1.34 [ 0.24;  7.59] | 8 | 10.80 [ 5.47;  21.31] | 2 | 2.74 [ 0.75;  9.99] | 3 | 4.14 [ 1.41;  12.17] |
| **Kanta-Hämeen** | 35 | 19.95 [ 14.35; 27.74] | 24 | 13.73 [ 9.23;  20.43] | 23 | 13.22 [ 8.81;  19.85] | 41 | 23.75 [ 17.51;  32.21] | 44 | 25.69 [ 19.14;  34.48] | 61 | 35.62 [ 27.74;  45.75] |
| **Keski-Pohjanmaan** | 12 | 15.90 [ 9.10;  27.79] | 13 | 16.54 [ 9.67;  28.30] | 17 | 21.65 [ 13.52;  34.67] | 24 | 30.72 [ 20.65;  45.71] | 36 | 46.32 [ 33.46;  64.12] | 24 | 30.99 [ 20.83;  46.11] |
| **Keski-Suomen** | 189 | 75.25 [ 65.26; 86.77] | 160 | 63.53 [ 54.42;  74.16] | 134 | 53.03 [ 44.78;  62.79] | 217 | 85.78 [ 75.10;  97.97] | 218 | 86.26 [ 75.55;  98.49] | 223 | 88.16 [ 77.33; 100.51] |
| **Kymenlaakson** | 167 | 96.59 [ 83.01; 112.38] | 182 | 105.90 [ 91.59; 122.43] | 226 | 132.18 [116.04; 150.56] | 206 | 122.11 [106.54; 139.95] | 258 | 154.85 [137.08; 174.91] | 260 | 157.54 [139.53; 177.87] |
| **Lapin** | 15 | 12.71 [ 7.70;  20.98] | 10 | 8.50 [ 4.62;  15.65] | 10 | 8.49 [ 4.61;  15.62] | 11 | 9.38 [ 5.24;  16.79] | 10 | 8.53 [ 4.63;  15.70] | 6 | 5.10 [ 2.34;  11.13] |
| **Länsi-Pohjan** | 3 | 4.72 [ 1.60;  13.87] | 1 | 1.58 [ 0.28;  8.98] | 5 | 8.00 [ 3.42;  18.73] | 4 | 6.48 [ 2.52;  16.66] | 4 | 6.54 [ 2.54;  16.81] | 6 | 9.93 [ 4.55;  21.66] |
| **Pirkanmaan** | 116 | 22.11 [ 18.43;  26.51] | 90 | 17.06 [ 13.88;  20.97] | 112 | 21.12 [ 17.55;  25.41] | 127 | 23.87 [ 20.06;  28.40] | 145 | 27.11 [ 23.04;  31.89] | 71 | 13.19 [ 10.46;  16.64] |
| **Pohjois-Karjalan** | 102 | 60.39 [ 49.76;  73.30] | 58 | 34.44 [ 26.64;  44.51] | 129 | 76.91 [ 64.74;  91.37] | 215 | 129.19 [113.05; 147.64] | 275 | 166.12 [147.63; 186.93] | 135 | 82.00 [ 69.29;  97.04] |
| **Pohjois-Pohjanmaan** | 23 | 5.64 [ 3.76;  8.46] | 12 | 2.94 [ 1.68;  5.15] | 29 | 7.10 [ 4.94;  10.19] | 29 | 7.08 [ 4.93;  10.17] | 142 | 34.68 [ 29.43;  40.88] | 96 | 23.40 [ 19.16;  28.57] |
| **Pohjois-Savon** | 198 | 80.93 [ 70.42;  93.01] | 138 | 55.62 [ 47.09;  65.71] | 158 | 64.97 [ 55.60;  75.91] | 259 | 105.00 [ 92.97; 118.58] | 243 | 98.94 [ 87.26; 112.18] | 77 | 31.47 [ 25.18;  39.32] |
| **Päijät-Hämeen** | 69 | 32.41 [ 25.61;  41.01] | 41 | 20.96 [ 15.45;  28.43] | 44 | 20.68 [ 15.41;  27.76] | 64 | 30.20 [ 23.66;  38.56] | 76 | 35.98 [ 28.75;  45.03] | 78 | 37.07 [ 29.71;  46.26] |
| **Satakunnan** | 48 | 21.61 [ 16.30;  28.65] | 55 | 24.96 [ 19.18;  32.48] | 52 | 23.80 [ 18.15;  31.20] | 70 | 31.76 [ 25.14;  40.12] | 62 | 28.35 [ 22.12;  36.34] | 85 | 39.17 [ 31.68;  48.43] |
| **Vaasan** | 66 | 38.90 [ 30.58;  49.49] | 55 | 32.30 [ 24.82;  42.03] | 56 | 32.91 [ 25.35;  42.73] | 70 | 41.24 [ 32.65;  52.10] | 60 | 35.36 [ 27.48;  45.51] | 36 | 21.20 [ 15.32;  29.35] |
| **Varsinais-Suomen** | 532 | 112.27 [103.13; 122.22] | 435 | 91.05 [ 82.89; 100.01] | 497 | 103.72 [ 95.00; 113.24] | 395 | 82.19 [ 74.47;  90.70] | 422 | 87.64 [ 79.67;  96.41] | 522 | 107.92 [ 99.06; 117.58] |
| **Total Finland** | 3,599 | 65.87 [ 63.75;  68.05] | 2,984 | 54.62 [ 52.69;  56.61] | 3,338 | 60.67 [ 58.65;  62.77] | 3,777 | 68.52 [ 66.37;  70.74] | 3,776 | 68.43 [ 66.28;  70.64] | 3,121 | 56.31 [ 54.37;  58.32] |
