# Supplementary material for: Incidence of Lyme Borreliosis in Finland: Exploring Observed Trends Over Time Using Public Surveillance Data, 2015–2020
Source: Vector Borne Zoonotic Dis. 2023 Apr 12;23(4):256–64. doi: 10.1089/vbz.2022.0047 (PMC10122252; doi:10.1089/vbz.2022.0047)
Supplement: Supplemental data [file Suppl_AppendixTableS3.docx]

**Table 3.** Number of cases (N) and incidence (per 100,000 residents, ± 95% CI) of both, clinically diagnosed and microbiologically confirmed Lyme borreliosis by year and Finnish municipalities, 2015–2020.

|  | **2015** | | **2016** | | **2017** | | **2018** | | **2019** | | **2020** | |
| --- | --- | --- | --- | --- | --- | --- | --- | --- | --- | --- | --- | --- |
| **HD/Municipality** | **N** | **Incidence [95% CI]** | **N** | **Incidence [95% CI]** | **N** | **Incidence [95% CI]** | **N** | **Incidence [95% CI]** | **N** | **Incidence [95% CI]** | **N** | **Incidence [95% CI]** |
| **Ahvenanmaa** |  |  |  |  |  |  |  |  |  |  |  |  |
| Brändö | 9 | 1,914.89 [1,010.64; 3,598.81] | 13 | 2,760.08 [1,619.98;  4,664.53] | 8 | 1,769.91 [ 899.51;  3,453.20] | 8 | 1,781.74 [ 905.54; 3,476.01] | 7 | 1,573.03 [ 764.02; 3,210.98] | 4 | 890.87 [ 346.97; 2,267.95] |
| Eckerö | 46 | 4,919.79 [3,708.53; 6,499.95] | 18 | 1,939.66 [1,230.39;  3,045.17] | 27 | 2,848.10 [1,964.66;  4,112.14] | 27 | 2,809.57 [1,937.98; 4,056.93] | 25 | 2,626.05 [1,784.98; 3,847.91] | 24 | 2,505.22 [1,689.19; 3,700.62] |
| Finström | 93 | 3,687.55 [3,019.67; 4,496.30] | 85 | 3,276.79 [2,657.81;  4,033.95] | 94 | 3,643.41 [2,986.60;  4,438.06] | 64 | 2,472.95 [1,941.38; 3,145.41] | 63 | 2,429.62 [1,903.64; 3,096.33] | 54 | 2,074.53 [1,593.47; 2,696.83] |
| Föglö | 9 | 1,624.55 [ 856.99; 3,058.36] | 10 | 1,782.53 [ 971.06;  3,249.85] | 18 | 3,383.46 [2,150.74;  5,284.57] | 10 | 1,872.66 [1,020.31; 3,412.49] | 8 | 1,506.59 [ 765.35; 2,944.44] | 6 | 1,140.68 [ 523.81; 2,466.04] |
| Geta | 19 | 3,800.00 [2,446.01; 5,858.48] | 25 | 5,010.02 [3,416.25;  7,291.19] | 29 | 5,858.59 [4,109.78;  8,287.24] | 22 | 4,280.16 [2,843.30; 6,395.33] | 7 | 1,411.29 [ 685.27; 2,884.15] | 14 | 2,739.73 [1,638.89; 4,545.82] |
| Hammarland | 59 | 3,838.65 [2,987.54; 4,919.92] | 67 | 4,442.97 [3,513.66;  5,603.79] | 53 | 3,425.99 [2,628.74;  4,453.96] | 39 | 2,473.05 [1,814.31; 3,362.77] | 33 | 2,084.65 [1,488.19; 2,913.10] | 34 | 2,126.33 [1,525.57; 2,956.56] |
| Jomala | 132 | 2,839.93 [2,399.94; 3,357.81] | 117 | 2,459.53 [2,056.25;  2,939.53] | 129 | 2,654.87 [2,238.89;  3,145.65] | 102 | 2,027.03 [1,672.69; 2,454.56] | 76 | 1,452.32 [1,161.97; 1,813.90] | 89 | 1,652.43 [1,344.82; 2,028.96] |
| Kökar | 11 | 4,400.00 [2,474.38; 7,705.78] | 9 | 3,658.54 [1,936.50;  6,805.63] | 17 | 7,203.39 [4,545.77; 11,231.93] | 11 | 4,661.02 [2,622.28; 8,152.11] | 3 | 1,293.10 [ 440.73; 3,732.18] | 9 | 4,000.00 [2,118.45; 7,425.91] |
| Kumlinge | 15 | 4,731.86 [2,888.24; 7,659.48] | 7 | 2,272.73 [1,105.18;  4,616.15] | 11 | 3,503.18 [1,967.18;  6,163.11] | 6 | 1,904.76 [ 875.82; 4,092.62] | 4 | 1,273.89 [ 496.47; 3,229.11] | 12 | 3,908.79 [2,249.90; 6,706.90] |
| Lemland | 53 | 2,661.98 [2,040.90; 3,465.37] | 58 | 2,882.70 [2,236.58;  3,708.40] | 65 | 3,205.13 [2,522.64;  4,064.56] | 49 | 2,410.23 [1,827.92; 3,172.05] | 44 | 2,143.21 [1,600.38; 2,864.79] | 56 | 2,649.01 [2,045.59; 3,424.20] |
| Lumparland | 14 | 3,517.59 [2,106.72; 5,817.17] | 17 | 4,415.58 [2,774.88;  6,956.96] | 13 | 3,291.14 [1,933.27;  5,548.76] | 10 | 2,617.80 [1,428.03; 4,751.05] | 6 | 1,639.34 [ 753.44; 3,529.87] | 7 | 1,881.72 [ 914.43; 3,832.64] |
| Maarianhamina | 301 | 2,626.30 [2,349.02; 2,935.32] | 265 | 2,291.40 [2,034.12;  2,580.36] | 287 | 2,457.82 [2,192.23;  2,754.68] | 216 | 1,839.39 [1,611.64; 2,098.65] | 177 | 1,515.54 [1,309.38; 1,753.59] | 215 | 1,836.82 [1,608.89; 2,096.36] |
| Saltvik | 56 | 3,061.78 [2,365.33; 3,954.99] | 68 | 3,697.66 [2,927.23;  4,661.13] | 82 | 4,378.00 [3,541.02;  5,401.74] | 48 | 2,583.42 [1,954.05; 3,408.46] | 37 | 2,001.08 [1,455.24; 2,745.95] | 39 | 2,159.47 [1,583.69; 2,938.34] |
| Sottunga | 3 | 3,030.30 [1,035.86; 8,533.68] | 4 | 4,166.67 [1,632.07; 10,228.19] | 2 | 2,173.91 [ 598.20;  7,583.49] | 2 | 2,197.80 [ 604.80; 7,663.16] | 0 | 0.00 [ 0.00; 4,182.71] | 1 | 990.10 [ 174.99; 5,396.72] |
| Sund | 41 | 3,976.72 [2,944.78; 5,350.35] | 40 | 3,976.14 [2,933.42;  5,369.02] | 36 | 3,491.76 [2,532.71;  4,796.09] | 32 | 3,112.84 [2,213.49; 4,361.31] | 25 | 2,443.79 [1,660.69; 3,582.71] | 18 | 1,787.49 [1,133.62; 2,807.80] |
| Vårdö | 16 | 3,628.12 [2,245.37; 5,811.76] | 15 | 3,416.86 [2,081.41;  5,560.48] | 30 | 6,976.74 [4,930.41;  9,784.98] | 12 | 2,678.57 [1,538.77; 4,623.00] | 8 | 1,789.71 [ 909.60; 3,491.38] | 4 | 869.57 [ 338.66; 2,214.25] |
| **Etelä-Karjalan** |  |  |  |  |  |  |  |  |  |  |  |  |
| Imatra | 42 | 150.89 [ 111.66;  203.88] | 32 | 116.29 [82.39;  164.12] | 34 | 124.68 [89.24;  174.17] | 37 | 137.38 [99.69;  189.30] | 33 | 124.49 [88.66;  174.77] | 40 | 153.40 [ 112.68;  208.81] |
| Lappeenranta | 221 | 303.26 [ 265.87;  345.88] | 167 | 229.17 [ 196.98;  266.61] | 149 | 204.36 [ 174.10;  239.87] | 191 | 262.73 [ 228.05;  302.66] | 141 | 194.12 [ 164.64;  228.88] | 179 | 246.35 [ 212.84;  285.12] |
| Lemi | 2 | 65.08 [17.85;  237.00] | 6 | 195.06 [89.43;  424.93] | 7 | 228.53 [ 110.75;  471.01] | 6 | 197.17 [90.40;  429.53] | 6 | 201.95 [92.59;  439.93] | 8 | 269.91 [ 136.83;  531.72] |
| Luumäki | 21 | 432.10 [ 282.80;  659.69] | 12 | 248.40 [ 142.15;  433.70] | 18 | 379.19 [ 239.99;  598.62] | 18 | 386.10 [ 244.37;  609.52] | 16 | 345.13 [ 212.55;  559.92] | 24 | 528.29 [ 355.27;  784.89] |
| Parikkala | 3 | 57.31 [19.49;  168.36] | 5 | 97.54 [41.67;  228.15] | 1 | 20.12 [ 3.55;  113.91] | 3 | 61.96 [21.07;  182.02] | 2 | 42.25 [11.59;  153.92] | 4 | 85.93 [33.42;  220.75] |
| Rautjärvi | 17 | 480.63 [ 300.31;  768.41] | 6 | 172.76 [79.20;  376.43] | 2 | 58.21 [15.96;  212.00] | 14 | 419.79 [ 250.23;  703.44] | 8 | 247.99 [ 125.71;  488.60] | 5 | 158.93 [67.90;  371.53] |
| Ruokolahti | 21 | 395.33 [ 258.72;  603.63] | 9 | 171.59 [90.30;  325.82] | 11 | 210.81 [ 117.76;  377.12] | 11 | 215.73 [ 120.50;  385.91] | 2 | 40.05 [10.98;  145.91] | 5 | 101.58 [43.40;  237.60] |
| Savitaipale | 3 | 83.03 [28.24;  243.86] | 7 | 198.08 [95.98;  408.32] | 1 | 28.74 [ 5.07;  162.60] | 3 | 87.49 [29.76;  256.93] | 3 | 88.68 [30.16;  260.42] | 3 | 90.20 [30.68;  264.87] |
| Taipalsaari | 7 | 145.38 [70.44;  299.80] | 12 | 248.34 [ 142.12;  433.61] | 12 | 251.36 [ 143.85;  438.87] | 8 | 169.67 [86.00;  334.47] | 4 | 85.63 [33.31;  220.00] | 16 | 345.72 [ 212.92;  560.88] |
| **Etelä-Savon** |  |  |  |  |  |  |  |  |  |  |  |  |
| Hirvensalmi | 0 | 0.00 [ 0.00;  167.47] | 2 | 87.95 [24.12;  320.13] | 0 | 0.00 [ 0.00;  171.51] | 1 | 46.47 [ 8.20;  262.76] | 4 | 187.27 [72.85;  480.53] | 1 | 46.38 [ 8.19;  262.27] |
| Joroinen | 3 | 58.71 [19.97;  172.48] | 4 | 79.38 [30.87;  203.94] | 5 | 101.69 [43.44;  237.84] | 5 | 103.91 [44.39;  243.02] | 4 | 83.91 [32.64;  215.57] | 0 | 0.00 [ 0.00;  81.86] |
| Juva | 8 | 122.17 [61.92;  240.92] | 7 | 109.02 [52.82;  224.88] | 5 | 78.94 [33.72;  184.67] | 5 | 80.32 [34.31;  187.90] | 6 | 98.10 [44.97;  213.88] | 10 | 168.58 [91.60;  310.06] |
| Kangasniemi | 7 | 124.38 [60.26;  256.53] | 3 | 53.54 [18.21;  157.31] | 5 | 90.11 [38.49;  210.77] | 1 | 18.34 [ 3.24;  103.83] | 1 | 18.67 [ 3.30;  105.69] | 5 | 94.13 [40.21;  220.17] |
| Mäntyharju | 4 | 64.95 [25.26;  166.88] | 1 | 16.40 [ 2.90;  92.85] | 3 | 49.55 [16.85;  145.60] | 4 | 67.52 [26.26;  173.50] | 2 | 34.54 [ 9.47;  125.85] | 1 | 17.62 [ 3.11;  99.74] |
| Mikkeli | 35 | 64.03 [46.04;  89.03] | 12 | 22.01 [12.59;  38.47] | 12 | 22.12 [12.65;  38.65] | 29 | 53.89 [37.52;  77.38] | 30 | 56.46 [39.55;  80.59] | 32 | 60.86 [43.11;  85.90] |
| Pertunmaa | 2 | 110.07 [30.19;  400.46] | 0 | 0.00 [ 0.00;  213.43] | 2 | 115.01 [31.55;  418.38] | 1 | 58.38 [10.31;  329.94] | 3 | 177.51 [60.39;  520.63] | 3 | 181.38 [61.70;  531.93] |
| Pieksämäki | 18 | 95.74 [60.57;  151.30] | 7 | 37.89 [18.35;  78.20] | 7 | 38.42 [18.61;  79.29] | 14 | 78.07 [46.51;  131.01] | 7 | 39.59 [19.18;  81.70] | 2 | 11.51 [ 3.16;  41.96] |
| Puumala | 7 | 309.73 [ 150.12;  637.99] | 4 | 179.05 [69.65;  459.50] | 0 | 0.00 [ 0.00;  173.68] | 7 | 318.62 [ 154.42;  656.24] | 8 | 371.92 [ 188.58;  732.21] | 6 | 280.77 [ 128.74;  611.22] |
| **Helsingin ja Uudenmaan** | |  |  |  |  |  |  |  |  |  |  |  |
| Askola | 3 | 58.78 [19.99;  172.68] | 0 | 0.00 [ 0.00;  76.07] | 2 | 40.08 [10.99;  146.03] | 4 | 80.68 [31.38;  207.27] | 2 | 40.46 [11.10;  147.42] | 2 | 41.00 [11.24;  149.38] |
| Espoo | 174 | 64.49 [55.60;  74.81] | 212 | 77.21 [67.49;  88.32] | 225 | 80.63 [70.77;  91.87] | 239 | 84.26 [74.24;  95.64] | 286 | 98.71 [87.92;  110.83] | 312 | 106.56 [95.38;  119.05] |
| Hanko | 58 | 654.33 [ 506.54;  844.87] | 37 | 427.10 [ 310.03;  588.12] | 37 | 434.43 [ 315.35;  598.19] | 32 | 381.91 [ 270.66;  538.63] | 27 | 329.31 [ 226.43;  478.71] | 25 | 310.87 [ 210.66;  458.52] |
| Helsinki | 834 | 132.76 [ 124.05;  142.07] | 799 | 125.79 [ 117.37;  134.81] | 913 | 141.93 [ 133.02;  151.43] | 1,007 | 155.39 [ 146.09;  165.28] | 1,015 | 155.24 [ 145.98;  165.08] | 527 | 80.22 [73.66;  87.37] |
| Hyvinkää | 15 | 32.28 [19.57;  53.26] | 11 | 23.61 [13.18;  42.27] | 14 | 29.95 [17.84;  50.28] | 17 | 36.56 [22.83;  58.54] | 14 | 30.13 [17.95;  50.57] | 13 | 27.91 [16.31;  47.75] |
| Inkoo | 26 | 469.23 [ 320.42;  686.66] | 14 | 250.67 [ 149.38;  420.35] | 19 | 346.65 [ 222.04;  540.81] | 24 | 444.20 [ 298.69;  660.12] | 8 | 148.53 [75.28;  292.84] | 45 | 845.71 [ 632.67; 1,129.67] |
| Järvenpää | 20 | 48.90 [31.66;  75.52] | 17 | 40.94 [25.56;  65.55] | 17 | 39.93 [24.93;  63.95] | 21 | 48.38 [31.64;  73.95] | 20 | 45.76 [29.62;  70.67] | 20 | 44.99 [29.13;  69.48] |
| Karkkila | 6 | 66.90 [30.66;  145.89] | 3 | 33.67 [11.45;  98.94] | 1 | 11.24 [ 1.98;  63.62] | 6 | 68.35 [31.33;  149.06] | 0 | 0.00 [ 0.00;  44.06] | 3 | 34.50 [11.73;  101.39] |
| Kauniainen | 40 | 421.67 [ 309.83;  573.65] | 35 | 372.46 [ 267.94;  517.54] | 39 | 405.24 [ 296.59;  553.46] | 32 | 332.81 [ 235.86;  469.44] | 17 | 173.52 [ 108.37;  277.73] | 18 | 176.85 [ 111.90;  279.40] |
| Kerava | 31 | 87.84 [61.89;  124.65] | 12 | 33.79 [19.33;  59.06] | 30 | 84.38 [59.11;  120.43] | 17 | 46.89 [29.28;  75.09] | 29 | 78.90 [54.94;  113.29] | 33 | 88.94 [63.34;  124.87] |
| Kirkkonummi | 163 | 421.74 [ 361.88;  491.46] | 165 | 422.72 [ 363.05;  492.14] | 144 | 367.63 [ 312.37;  432.62] | 173 | 440.63 [ 379.79;  511.17] | 204 | 515.33 [ 449.44;  590.83] | 159 | 396.69 [ 339.72;  463.16] |
| Lapinjärvi | 1 | 36.05 [ 6.36;  203.92] | 0 | 0.00 [ 0.00;  140.05] | 2 | 73.91 [20.27;  269.10] | 3 | 112.57 [38.29;  330.46] | 1 | 38.37 [ 6.77;  217.05] | 1 | 38.15 [ 6.74;  215.81] |
| Lohja | 65 | 137.27 [ 107.72;  174.90] | 46 | 97.56 [73.16;  130.10] | 53 | 113.28 [86.63;  148.13] | 57 | 123.12 [95.05;  159.47] | 73 | 158.82 [ 126.34;  199.62] | 72 | 156.91 [ 124.63;  197.54] |
| Loviisa | 21 | 137.16 [89.73;  209.60] | 18 | 118.36 [74.88;  187.03] | 17 | 112.69 [70.38;  180.42] | 27 | 181.32 [ 124.65;  263.69] | 21 | 142.16 [93.00;  217.24] | 27 | 183.11 [ 125.88;  266.29] |
| Mäntsälä | 5 | 24.17 [10.33;  56.58] | 6 | 28.77 [13.19;  62.77] | 4 | 19.23 [ 7.48;  49.43] | 2 | 9.67 [ 2.65;  35.25] | 10 | 48.26 [26.22;  88.82] | 3 | 14.43 [ 4.91;  42.44] |
| Nurmijärvi | 44 | 105.02 [78.25;  140.94] | 32 | 76.17 [53.96;  107.51] | 53 | 125.71 [96.13;  164.38] | 62 | 145.32 [ 113.38;  186.23] | 58 | 134.91 [ 104.38;  174.34] | 49 | 112.22 [84.90;  148.32] |
| Pornainen | 0 | 0.00 [ 0.00;  74.90] | 0 | 0.00 [ 0.00;  75.15] | 0 | 0.00 [ 0.00;  74.96] | 2 | 39.46 [10.82;  143.78] | 1 | 19.86 [ 3.51;  112.42] | 0 | 0.00 [ 0.00;  75.71] |
| Porvoo | 46 | 92.13 [69.09;  122.86] | 49 | 97.72 [73.93;  129.15] | 47 | 93.70 [70.48;  124.57] | 70 | 139.27 [ 110.26;  175.90] | 74 | 146.88 [ 117.03;  184.34] | 78 | 154.09 [ 123.50;  192.25] |
| Raasepori | 62 | 218.27 [ 170.32;  279.69] | 58 | 206.57 [ 159.85;  266.93] | 79 | 283.65 [ 227.68;  353.34] | 81 | 293.56 [ 236.28;  364.69] | 80 | 290.53 [ 233.52;  361.41] | 76 | 276.08 [ 220.65;  345.39] |
| Sipoo | 19 | 97.94 [62.71;  152.93] | 13 | 65.25 [38.14;  111.62] | 15 | 73.86 [44.76;  121.83] | 23 | 111.29 [74.18;  166.96] | 23 | 108.64 [72.41;  162.98] | 16 | 73.78 [45.42;  119.82] |
| Siuntio | 12 | 194.11 [ 111.08;  339.01] | 18 | 291.36 [ 184.38;  460.11] | 15 | 244.06 [ 147.96;  402.32] | 13 | 211.93 [ 123.90;  362.29] | 21 | 341.74 [ 223.64;  521.89] | 15 | 243.94 [ 147.89;  402.12] |
| Tuusula | 21 | 54.60 [35.72;  83.47] | 29 | 75.15 [52.33;  107.91] | 31 | 80.22 [56.52;  113.83] | 36 | 93.11 [67.27;  128.87] | 35 | 90.68 [65.21;  126.08] | 18 | 46.41 [29.36;  73.36] |
| Vantaa | 166 | 77.35 [66.45;  90.04] | 163 | 74.31 [63.75;  86.63] | 216 | 96.85 [84.77;  110.65] | 214 | 93.79 [82.04;  107.22] | 100 | 42.78 [35.17;  52.02] | 115 | 48.48 [40.39;  58.18] |
| Vihti | 10 | 34.58 [18.78;  63.65] | 15 | 51.78 [31.38;  85.43] | 22 | 75.72 [50.01;  114.63] | 10 | 34.23 [18.60;  63.01] | 14 | 48.01 [28.60;  80.58] | 29 | 99.45 [69.26;  142.79] |
| **Itä-Savon** |  |  |  |  |  |  |  |  |  |  |  |  |
| Enonkoski | 0 | 0.00 [ 0.00;  260.11] | 3 | 206.47 [70.24;  605.29] | 0 | 0.00 [ 0.00;  270.56] | 1 | 71.17 [12.57;  402.06] | 1 | 73.48 [12.97;  415.02] | 1 | 73.05 [12.90;  412.61] |
| Rantasalmi | 0 | 0.00 [ 0.00;  102.80] | 1 | 27.40 [ 4.84;  155.08] | 0 | 0.00 [ 0.00;  108.00] | 1 | 28.46 [ 5.02;  161.03] | 4 | 116.58 [45.35;  299.40] | 7 | 208.09 [ 100.83;  428.93] |
| Savonlinna | 30 | 84.45 [59.17;  120.53] | 26 | 73.78 [50.35;  108.08] | 26 | 75.01 [51.19;  109.88] | 40 | 119.01 [87.41;  162.00] | 35 | 106.14 [76.33;  147.58] | 30 | 91.85 [64.35;  131.09] |
| Sulkava | 7 | 256.98 [ 124.53;  529.52] | 1 | 37.58 [ 6.63;  212.57] | 1 | 38.64 [ 6.82;  218.56] | 0 | 0.00 [ 0.00;  151.61] | 0 | 0.00 [ 0.00;  153.91] | 0 | 0.00 [ 0.00;  154.53] |
| **Kainuun** |  |  |  |  |  |  |  |  |  |  |  |  |
| Hyrynsalmi | 0 | 0.00 [ 0.00;  158.36] | 0 | 0.00 [ 0.00;  159.41] | 0 | 0.00 [ 0.00;  164.88] | 0 | 0.00 [ 0.00;  167.69] | 0 | 0.00 [ 0.00;  168.87] | 0 | 0.00 [ 0.00;  174.39] |
| Kajaani | 3 | 7.97 [ 2.71;  23.44] | 7 | 18.66 [ 9.04;  38.51] | 1 | 2.69 [ 0.47;  15.21] | 4 | 10.82 [ 4.21;  27.82] | 0 | 0.00 [ 0.00;  10.46] | 2 | 5.47 [ 1.50;  19.94] |
| Kuhmo | 2 | 22.71 [ 6.23;  82.78] | 0 | 0.00 [ 0.00;  44.41] | 1 | 11.77 [ 2.08;  66.62] | 4 | 48.02 [18.68;  123.43] | 0 | 0.00 [ 0.00;  46.88] | 0 | 0.00 [ 0.00;  47.74] |
| Paltamo | 0 | 0.00 [ 0.00;  110.01] | 0 | 0.00 [ 0.00;  109.92] | 0 | 0.00 [ 0.00;  111.71] | 0 | 0.00 [ 0.00;  115.02] | 1 | 30.55 [ 5.39;  172.87] | 1 | 30.91 [ 5.46;  174.90] |
| Puolanka | 0 | 0.00 [ 0.00;  138.19] | 1 | 36.56 [ 6.45;  206.83] | 0 | 0.00 [ 0.00;  143.72] | 0 | 0.00 [ 0.00;  147.70] | 0 | 0.00 [ 0.00;  151.73] | 1 | 40.14 [ 7.09;  227.05] |
| Ristijärvi | 0 | 0.00 [ 0.00;  283.54] | 0 | 0.00 [ 0.00;  284.80] | 0 | 0.00 [ 0.00;  290.83] | 0 | 0.00 [ 0.00;  297.36] | 0 | 0.00 [ 0.00;  301.09] | 0 | 0.00 [ 0.00;  310.08] |
| Sotkamo | 2 | 19.01 [ 5.21;  69.28] | 1 | 9.55 [ 1.69;  54.08] | 0 | 0.00 [ 0.00;  36.84] | 1 | 9.63 [ 1.70;  54.51] | 2 | 19.35 [ 5.31;  70.53] | 1 | 9.71 [ 1.71;  54.97] |
| Suomussalmi | 1 | 12.00 [ 2.12;  67.93] | 0 | 0.00 [ 0.00;  46.90] | 0 | 0.00 [ 0.00;  47.69] | 0 | 0.00 [ 0.00;  48.84] | 1 | 12.94 [ 2.28;  73.28] | 0 | 0.00 [ 0.00;  50.56] |
| **Kanta-Hämeen** |  |  |  |  |  |  |  |  |  |  |  |  |
| Forssa | 3 | 17.22 [ 5.86;  50.62] | 9 | 51.93 [27.32;  98.67] | 3 | 17.46 [ 5.94;  51.32] | 8 | 46.98 [23.81;  92.69] | 4 | 23.67 [ 9.20;  60.84] | 13 | 77.38 [45.23;  132.36] |
| Hämeenlinna | 25 | 36.76 [24.90;  54.26] | 15 | 22.11 [13.40;  36.48] | 16 | 23.65 [14.56;  38.41] | 23 | 34.06 [22.70;  51.10] | 15 | 22.18 [13.44;  36.59] | 32 | 47.16 [33.41;  66.57] |
| Hattula | 0 | 0.00 [ 0.00;  39.40] | 0 | 0.00 [ 0.00;  39.66] | 2 | 20.81 [ 5.71;  75.86] | 3 | 31.66 [10.77;  93.06] | 9 | 95.52 [50.26;  181.46] | 5 | 53.25 [22.75;  124.61] |
| Hausjärvi | 1 | 11.46 [ 2.02;  64.87] | 2 | 23.15 [ 6.35;  84.36] | 1 | 11.76 [ 2.08;  66.58] | 5 | 59.40 [25.38;  139.00] | 2 | 24.21 [ 6.64;  88.25] | 5 | 61.16 [26.13;  143.11] |
| Humppila | 1 | 41.88 [ 7.39;  236.83] | 0 | 0.00 [ 0.00;  163.55] | 0 | 0.00 [ 0.00;  167.47] | 0 | 0.00 [ 0.00;  171.58] | 1 | 45.79 [ 8.08;  258.91] | 0 | 0.00 [ 0.00;  176.39] |
| Janakkala | 1 | 5.93 [ 1.05;  33.61] | 6 | 35.91 [16.46;  78.33] | 3 | 18.06 [ 6.14;  53.10] | 5 | 30.40 [12.99;  71.15] | 9 | 54.83 [28.85;  104.19] | 5 | 30.79 [13.15;  72.07] |
| Jokioinen | 0 | 0.00 [ 0.00;  70.76] | 0 | 0.00 [ 0.00;  71.87] | 1 | 18.92 [ 3.34;  107.09] | 1 | 19.25 [ 3.40;  108.96] | 3 | 58.45 [19.88;  171.71] | 0 | 0.00 [ 0.00;  75.85] |
| Loppi | 4 | 48.93 [19.03;  125.75] | 1 | 12.35 [ 2.18;  69.92] | 0 | 0.00 [ 0.00;  47.83] | 3 | 38.16 [12.98;  112.15] | 3 | 38.32 [13.03;  112.63] | 2 | 25.47 [ 6.98;  92.82] |
| Riihimäki | 9 | 30.75 [16.18;  58.43] | 6 | 20.58 [ 9.43;  44.89] | 10 | 34.46 [18.72;  63.42] | 17 | 59.16 [36.94;  94.73] | 13 | 45.15 [26.39;  77.24] | 15 | 52.25 [31.67;  86.19] |
| Tammela | 0 | 0.00 [ 0.00;  61.13] | 0 | 0.00 [ 0.00;  61.51] | 2 | 32.49 [ 8.91;  118.41] | 0 | 0.00 [ 0.00;  63.13] | 0 | 0.00 [ 0.00;  63.82] | 3 | 49.87 [16.96;  146.52] |
| Ypäjä | 0 | 0.00 [ 0.00;  159.08] | 0 | 0.00 [ 0.00;  161.01] | 1 | 42.16 [ 7.44;  238.43] | 0 | 0.00 [ 0.00;  162.72] | 1 | 42.68 [ 7.53;  241.37] | 1 | 43.22 [ 7.63;  244.39] |
| **Keski-Pohjanmaan** |  |  |  |  |  |  |  |  |  |  |  |  |
| Halsua | 0 | 0.00 [ 0.00;  312.61] | 0 | 0.00 [ 0.00;  314.14] | 0 | 0.00 [ 0.00;  326.98] | 0 | 0.00 [ 0.00;  328.66] | 0 | 0.00 [ 0.00;  339.70] | 0 | 0.00 [ 0.00;  347.06] |
| Kannus | 1 | 17.89 [ 3.16;  101.27] | 0 | 0.00 [ 0.00;  68.82] | 0 | 0.00 [ 0.00;  69.54] | 3 | 54.53 [18.55;  160.20] | 1 | 18.30 [ 3.23;  103.60] | 1 | 18.43 [ 3.25;  104.33] |
| Kaustinen | 1 | 23.23 [ 4.10;  131.47] | 1 | 23.27 [ 4.11;  131.68] | 0 | 0.00 [ 0.00;  89.07] | 0 | 0.00 [ 0.00;  89.82] | 3 | 70.41 [23.95;  206.81] | 3 | 70.96 [24.13;  208.42] |
| Kokkola | 17 | 35.74 [22.31;  57.23] | 14 | 29.34 [17.48;  49.24] | 22 | 46.10 [30.45;  69.79] | 23 | 48.26 [32.16;  72.41] | 34 | 71.31 [51.04;  99.62] | 24 | 50.24 [33.76;  74.75] |
| Kruunupyy | 2 | 29.93 [ 8.21;  109.08] | 2 | 30.21 [ 8.29;  110.10] | 3 | 45.93 [15.62;  134.98] | 5 | 76.82 [32.82;  179.71] | 1 | 15.56 [ 2.75;  88.07] | 2 | 31.17 [ 8.55;  113.60] |
| Lestijärvi | 0 | 0.00 [ 0.00;  479.08] | 0 | 0.00 [ 0.00;  471.44] | 0 | 0.00 [ 0.00;  484.52] | 0 | 0.00 [ 0.00;  518.53] | 0 | 0.00 [ 0.00;  531.44] | 0 | 0.00 [ 0.00;  529.24] |
| Perho | 0 | 0.00 [ 0.00;  130.89] | 0 | 0.00 [ 0.00;  131.97] | 0 | 0.00 [ 0.00;  134.14] | 0 | 0.00 [ 0.00;  135.80] | 0 | 0.00 [ 0.00;  139.04] | 0 | 0.00 [ 0.00;  141.76] |
| Reisjärvi | 0 | 0.00 [ 0.00;  132.56] | 0 | 0.00 [ 0.00;  134.42] | 0 | 0.00 [ 0.00;  136.37] | 0 | 0.00 [ 0.00;  139.85] | 0 | 0.00 [ 0.00;  141.13] | 0 | 0.00 [ 0.00;  141.55] |
| Toholampi | 0 | 0.00 [ 0.00;  115.89] | 0 | 0.00 [ 0.00;  118.72] | 0 | 0.00 [ 0.00;  120.20] | 1 | 32.13 [ 5.67;  181.80] | 2 | 65.94 [18.09;  240.13] | 0 | 0.00 [ 0.00;  129.35] |
| Veteli | 3 | 90.85 [30.90;  266.80] | 0 | 0.00 [ 0.00;  117.73] | 1 | 31.09 [ 5.49;  175.93] | 0 | 0.00 [ 0.00;  121.19] | 1 | 32.11 [ 5.67;  181.69] | 2 | 65.25 [17.90;  237.62] |
| **Keski-Suomen** |  |  |  |  |  |  |  |  |  |  |  |  |
| Äänekoski | 25 | 127.25 [86.21;  187.79] | 30 | 154.85 [ 108.49;  220.96] | 21 | 109.69 [71.76;  167.65] | 32 | 169.75 [ 120.27;  239.53] | 28 | 149.21 [ 103.26;  215.57] | 19 | 102.28 [65.49;  159.70] |
| Hankasalmi | 5 | 95.42 [40.76;  223.19] | 5 | 96.92 [41.40;  226.69] | 2 | 39.85 [10.93;  145.19] | 3 | 60.74 [20.66;  178.45] | 8 | 164.10 [83.18;  323.51] | 4 | 83.65 [32.53;  214.89] |
| Joutsa | 2 | 42.66 [11.70;  155.43] | 2 | 42.80 [11.74;  155.93] | 3 | 65.69 [22.34;  192.97] | 2 | 44.77 [12.28;  163.11] | 1 | 22.85 [ 4.03;  129.31] | 3 | 69.82 [23.75;  205.08] |
| Jyväskylä | 163 | 118.66 [ 101.80;  138.31] | 136 | 97.95 [82.82;  115.84] | 116 | 82.75 [69.00;  99.23] | 185 | 130.92 [ 113.38;  151.18] | 201 | 141.15 [ 122.95;  162.04] | 186 | 129.69 [ 112.35;  149.70] |
| Kannonkoski | 2 | 136.80 [37.52;  497.42] | 0 | 0.00 [ 0.00;  269.04] | 0 | 0.00 [ 0.00;  272.09] | 1 | 73.91 [13.05;  417.47] | 0 | 0.00 [ 0.00;  286.07] | 0 | 0.00 [ 0.00;  289.52] |
| Karstula | 0 | 0.00 [ 0.00;  89.93] | 1 | 23.63 [ 4.17;  133.73] | 0 | 0.00 [ 0.00;  92.57] | 0 | 0.00 [ 0.00;  95.21] | 2 | 50.65 [13.89;  184.49] | 0 | 0.00 [ 0.00;  99.47] |
| Keuruu | 2 | 19.77 [ 5.42;  72.06] | 0 | 0.00 [ 0.00;  38.43] | 6 | 60.49 [27.73;  131.92] | 0 | 0.00 [ 0.00;  39.34] | 0 | 0.00 [ 0.00;  39.98] | 4 | 42.17 [16.40;  108.38] |
| Kinnula | 0 | 0.00 [ 0.00;  219.66] | 0 | 0.00 [ 0.00;  225.59] | 0 | 0.00 [ 0.00;  231.44] | 0 | 0.00 [ 0.00;  237.30] | 0 | 0.00 [ 0.00;  236.57] | 0 | 0.00 [ 0.00;  239.96] |
| Kivijärvi | 0 | 0.00 [ 0.00;  319.10] | 1 | 86.13 [15.21;  486.27] | 0 | 0.00 [ 0.00;  338.20] | 0 | 0.00 [ 0.00;  347.06] | 0 | 0.00 [ 0.00;  349.27] | 0 | 0.00 [ 0.00;  345.82] |
| Konnevesi | 1 | 36.27 [ 6.40;  205.18] | 0 | 0.00 [ 0.00;  139.34] | 1 | 36.39 [ 6.42;  205.85] | 0 | 0.00 [ 0.00;  142.18] | 1 | 38.07 [ 6.72;  215.32] | 0 | 0.00 [ 0.00;  147.93] |
| Kyyjärvi | 0 | 0.00 [ 0.00;  277.79] | 0 | 0.00 [ 0.00;  278.60] | 0 | 0.00 [ 0.00;  283.33] | 0 | 0.00 [ 0.00;  285.22] | 0 | 0.00 [ 0.00;  291.72] | 0 | 0.00 [ 0.00;  297.36] |
| Laukaa | 4 | 21.20 [ 8.25;  54.51] | 2 | 10.54 [ 2.89;  38.44] | 1 | 5.27 [ 0.93;  29.84] | 4 | 21.13 [ 8.22;  54.33] | 6 | 31.74 [14.55;  69.24] | 4 | 21.25 [ 8.26;  54.63] |
| Luhanka | 1 | 131.41 [23.20;  740.55] | 0 | 0.00 [ 0.00;  505.56] | 0 | 0.00 [ 0.00;  520.63] | 1 | 141.44 [24.97;  796.79] | 0 | 0.00 [ 0.00;  553.65] | 0 | 0.00 [ 0.00;  546.56] |
| Multia | 0 | 0.00 [ 0.00;  224.14] | 1 | 60.13 [10.62;  339.84] | 0 | 0.00 [ 0.00;  234.26] | 0 | 0.00 [ 0.00;  241.93] | 0 | 0.00 [ 0.00;  244.70] | 0 | 0.00 [ 0.00;  245.96] |
| Muurame | 17 | 173.63 [ 108.44;  277.90] | 9 | 90.53 [47.64;  171.99] | 11 | 108.94 [60.84;  194.99] | 22 | 216.32 [ 142.90;  327.34] | 23 | 226.29 [ 150.84;  339.35] | 30 | 292.20 [ 204.76;  416.82] |
| Petäjävesi | 0 | 0.00 [ 0.00;  95.75] | 0 | 0.00 [ 0.00;  96.40] | 2 | 51.02 [13.99;  185.85] | 0 | 0.00 [ 0.00;  98.40] | 1 | 26.03 [ 4.60;  147.33] | 0 | 0.00 [ 0.00;  101.74] |
| Pihtipudas | 0 | 0.00 [ 0.00;  90.93] | 1 | 23.80 [ 4.20;  134.69] | 0 | 0.00 [ 0.00;  92.99] | 1 | 24.67 [ 4.36;  139.63] | 1 | 24.80 [ 4.38;  140.36] | 1 | 25.44 [ 4.49;  143.96] |
| Saarijärvi | 0 | 0.00 [ 0.00;  38.73] | 1 | 10.32 [ 1.82;  58.44] | 4 | 41.71 [16.22;  107.22] | 0 | 0.00 [ 0.00;  40.78] | 0 | 0.00 [ 0.00;  41.25] | 0 | 0.00 [ 0.00;  41.70] |
| Toivakka | 2 | 82.27 [22.56;  299.49] | 0 | 0.00 [ 0.00;  157.71] | 0 | 0.00 [ 0.00;  160.88] | 0 | 0.00 [ 0.00;  159.41] | 1 | 41.88 [ 7.39;  236.83] | 0 | 0.00 [ 0.00;  159.74] |
| Uurainen | 1 | 27.28 [ 4.82;  154.36] | 1 | 26.90 [ 4.75;  152.24] | 0 | 0.00 [ 0.00;  102.42] | 0 | 0.00 [ 0.00;  101.44] | 1 | 27.17 [ 4.80;  153.73] | 0 | 0.00 [ 0.00;  105.25] |
| Viitasaari | 2 | 30.00 [ 8.23;  109.34] | 4 | 60.54 [23.55;  155.58] | 2 | 31.20 [ 8.56;  113.68] | 2 | 31.93 [ 8.76;  116.35] | 2 | 32.38 [ 8.88;  118.01] | 2 | 32.80 [ 9.00;  119.53] |
| **Kymenlaakson** |  |  |  |  |  |  |  |  |  |  |  |  |
| Hamina | 92 | 441.23 [ 359.94;  540.77] | 87 | 421.59 [ 341.95;  519.70] | 93 | 453.81 [ 370.62;  555.58] | 90 | 443.66 [ 361.11;  544.96] | 81 | 402.76 [ 324.20;  500.27] | 106 | 533.28 [ 441.15;  644.53] |
| Kotka | 89 | 163.85 [ 133.18;  201.56] | 115 | 212.23 [ 176.85;  254.66] | 159 | 296.98 [ 254.32;  346.77] | 145 | 274.19 [ 233.09;  322.51] | 223 | 427.81 [ 375.32;  487.61] | 153 | 296.12 [ 252.82;  346.81] |
| Kouvola | 50 | 58.24 [44.18;  76.76] | 47 | 55.10 [41.44;  73.25] | 44 | 52.26 [38.93;  70.14] | 30 | 36.07 [25.27;  51.48] | 52 | 63.33 [48.30;  83.03] | 57 | 70.21 [54.20;  90.94] |
| Miehikkälä | 3 | 143.88 [48.95;  422.20] | 1 | 49.16 [ 8.68;  277.97] | 0 | 0.00 [ 0.00;  192.47] | 2 | 103.09 [28.28;  375.12] | 1 | 53.85 [ 9.51;  304.41] | 3 | 160.60 [54.63;  471.13] |
| Pyhtää | 2 | 37.59 [10.31;  136.95] | 5 | 93.63 [40.00;  219.02] | 4 | 75.99 [29.55;  195.23] | 2 | 38.56 [10.57;  140.49] | 2 | 38.91 [10.67;  141.77] | 3 | 58.54 [19.91;  171.97] |
| Virolahti | 10 | 298.78 [ 162.37;  549.13] | 3 | 91.83 [31.23;  269.65] | 8 | 249.45 [ 126.46;  491.49] | 9 | 285.71 [ 150.39;  542.15] | 10 | 321.65 [ 174.81;  591.10] | 9 | 291.55 [ 153.46;  553.19] |
| **Länsi-Pohjan** |  |  |  |  |  |  |  |  |  |  |  |  |
| Kemi | 4 | 18.38 [ 7.15;  47.26] | 1 | 4.63 [ 0.82;  26.22] | 4 | 18.82 [ 7.32;  48.38] | 6 | 28.54 [13.08;  62.26] | 5 | 24.15 [10.31;  56.52] | 2 | 9.79 [ 2.68;  35.68] |
| Keminmaa | 1 | 11.92 [ 2.10;  67.50] | 0 | 0.00 [ 0.00;  46.17] | 0 | 0.00 [ 0.00;  46.28] | 0 | 0.00 [ 0.00;  47.13] | 1 | 12.38 [ 2.19;  70.08] | 0 | 0.00 [ 0.00;  48.09] |
| Simo | 1 | 30.88 [ 5.45;  174.74] | 0 | 0.00 [ 0.00;  121.03] | 2 | 64.31 [17.64;  234.19] | 1 | 32.84 [ 5.80;  185.80] | 0 | 0.00 [ 0.00;  128.40] | 5 | 169.49 [72.42;  396.17] |
| Tervola | 0 | 0.00 [ 0.00;  120.09] | 0 | 0.00 [ 0.00;  123.80] | 1 | 32.59 [ 5.75;  184.41] | 1 | 32.66 [ 5.77;  184.77] | 0 | 0.00 [ 0.00;  127.84] | 0 | 0.00 [ 0.00;  131.16] |
| Tornio | 0 | 0.00 [ 0.00;  17.30] | 2 | 9.04 [ 2.48;  32.97] | 1 | 4.56 [ 0.81;  25.83] | 0 | 0.00 [ 0.00;  17.56] | 3 | 13.89 [ 4.72;  40.83] | 1 | 4.66 [ 0.82;  26.38] |
| Ylitornio | 1 | 23.30 [ 4.11;  131.90] | 0 | 0.00 [ 0.00;  91.38] | 0 | 0.00 [ 0.00;  93.20] | 0 | 0.00 [ 0.00;  95.42] | 0 | 0.00 [ 0.00;  97.95] | 0 | 0.00 [ 0.00;  98.65] |
| **Lapin** |  |  |  |  |  |  |  |  |  |  |  |  |
| Enontekiö | 0 | 0.00 [ 0.00;  205.99] | 0 | 0.00 [ 0.00;  204.79] | 0 | 0.00 [ 0.00;  202.52] | 0 | 0.00 [ 0.00;  206.99] | 0 | 0.00 [ 0.00;  208.57] | 0 | 0.00 [ 0.00;  212.02] |
| Inari | 0 | 0.00 [ 0.00;  56.43] | 0 | 0.00 [ 0.00;  56.25] | 0 | 0.00 [ 0.00;  55.89] | 0 | 0.00 [ 0.00;  55.40] | 0 | 0.00 [ 0.00;  55.59] | 0 | 0.00 [ 0.00;  55.95] |
| Kemijärvi | 1 | 12.88 [ 2.27;  72.91] | 1 | 13.05 [ 2.30;  73.91] | 1 | 13.27 [ 2.34;  75.15] | 0 | 0.00 [ 0.00;  52.10] | 1 | 13.75 [ 2.43;  77.84] | 0 | 0.00 [ 0.00;  53.39] |
| Kittilä | 1 | 15.59 [ 2.75;  88.24] | 1 | 15.67 [ 2.77;  88.70] | 0 | 0.00 [ 0.00;  59.79] | 0 | 0.00 [ 0.00;  59.65] | 1 | 15.50 [ 2.74;  87.73] | 1 | 15.54 [ 2.74;  87.97] |
| Kolari | 0 | 0.00 [ 0.00;  99.73] | 0 | 0.00 [ 0.00;  100.28] | 0 | 0.00 [ 0.00;  99.58] | 1 | 26.08 [ 4.60;  147.60] | 0 | 0.00 [ 0.00;  99.78] | 0 | 0.00 [ 0.00;  97.78] |
| Muonio | 1 | 42.41 [ 7.49;  239.84] | 0 | 0.00 [ 0.00;  163.20] | 1 | 42.88 [ 7.57;  242.51] | 0 | 0.00 [ 0.00;  166.81] | 0 | 0.00 [ 0.00;  166.16] | 0 | 0.00 [ 0.00;  166.96] |
| Pelkosenniemi | 0 | 0.00 [ 0.00;  399.39] | 0 | 0.00 [ 0.00;  402.31] | 0 | 0.00 [ 0.00;  399.39] | 0 | 0.00 [ 0.00;  401.05] | 0 | 0.00 [ 0.00;  407.43] | 0 | 0.00 [ 0.00;  410.92] |
| Pello | 0 | 0.00 [ 0.00;  105.92] | 1 | 28.05 [ 4.95;  158.73] | 1 | 28.49 [ 5.03;  161.21] | 1 | 29.09 [ 5.13;  164.58] | 1 | 29.65 [ 5.23;  167.75] | 1 | 30.27 [ 5.34;  171.25] |
| Posio | 0 | 0.00 [ 0.00;  110.36] | 1 | 29.21 [ 5.16;  165.26] | 0 | 0.00 [ 0.00;  115.92] | 0 | 0.00 [ 0.00;  118.53] | 0 | 0.00 [ 0.00;  120.54] | 0 | 0.00 [ 0.00;  123.09] |
| Ranua | 0 | 0.00 [ 0.00;  95.47] | 0 | 0.00 [ 0.00;  95.40] | 1 | 25.18 [ 4.44;  142.48] | 0 | 0.00 [ 0.00;  98.50] | 1 | 26.43 [ 4.67;  149.59] | 1 | 26.94 [ 4.76;  152.45] |
| Rovaniemi | 13 | 21.02 [12.29;  35.97] | 6 | 9.64 [ 4.42;  21.04] | 12 | 19.22 [11.00;  33.60] | 12 | 19.07 [10.91;  33.33] | 12 | 19.03 [10.89;  33.27] | 2 | 3.15 [ 0.86;  11.48] |
| Salla | 1 | 26.83 [ 4.74;  151.84] | 0 | 0.00 [ 0.00;  105.05] | 0 | 0.00 [ 0.00;  107.34] | 1 | 28.65 [ 5.06;  162.09] | 0 | 0.00 [ 0.00;  112.86] | 1 | 29.35 [ 5.18;  166.08] |
| Savukoski | 0 | 0.00 [ 0.00;  360.75] | 0 | 0.00 [ 0.00;  366.61] | 1 | 98.81 [17.45;  557.59] | 0 | 0.00 [ 0.00;  377.04] | 0 | 0.00 [ 0.00;  380.78] | 0 | 0.00 [ 0.00;  379.28] |
| Sodankylä | 2 | 22.77 [ 6.25;  83.01] | 2 | 23.11 [ 6.34;  84.24] | 0 | 0.00 [ 0.00;  44.94] | 0 | 0.00 [ 0.00;  45.47] | 1 | 12.04 [ 2.13;  68.19] | 0 | 0.00 [ 0.00;  46.45] |
| Utsjoki | 0 | 0.00 [ 0.00;  306.38] | 1 | 80.58 [14.23;  455.03] | 0 | 0.00 [ 0.00;  308.34] | 0 | 0.00 [ 0.00;  310.84] | 0 | 0.00 [ 0.00;  315.95] | 1 | 82.03 [14.48;  463.21] |
| **Päijät-Hämeen** |  |  |  |  |  |  |  |  |  |  |  |  |
| Asikkala | 8 | 96.54 [48.93;  190.39] | 4 | 48.06 [18.69;  123.52] | 2 | 24.25 [ 6.65;  88.38] | 7 | 85.90 [41.62;  177.22] | 12 | 148.46 [84.95;  259.33] | 3 | 37.23 [12.66;  109.40] |
| Hartola | 1 | 33.53 [ 5.92;  189.72] | 1 | 34.20 [ 6.04;  193.48] | 0 | 0.00 [ 0.00;  133.11] | 2 | 71.94 [19.73;  261.95] | 0 | 0.00 [ 0.00;  142.23] | 0 | 0.00 [ 0.00;  144.48] |
| Heinola | 15 | 76.63 [46.45;  126.40] | 12 | 62.02 [35.48;  108.37] | 14 | 73.19 [43.61;  122.83] | 17 | 90.00 [56.20;  144.09] | 25 | 133.93 [90.73;  197.64] | 20 | 108.13 [70.01;  166.96] |
| Hollola | 9 | 37.63 [19.80;  71.51] | 18 | 75.66 [47.87;  119.57] | 8 | 33.64 [17.05;  66.37] | 3 | 12.71 [ 4.32;  37.37] | 10 | 42.72 [23.21;  78.62] | 9 | 38.71 [20.37;  73.56] |
| Iitti | 4 | 57.89 [22.51;  148.76] | 2 | 29.03 [ 7.96;  105.80] | 5 | 73.31 [31.32;  171.52] | 7 | 103.47 [50.13;  213.45] | 3 | 44.70 [15.20;  131.36] | 5 | 75.47 [32.24;  176.57] |
| Kärkölä | 3 | 65.16 [22.16;  191.42] | 0 | 0.00 [ 0.00;  84.54] | 3 | 66.55 [22.63;  195.49] | 0 | 0.00 [ 0.00;  86.23] | 6 | 137.36 [62.97;  299.38] | 1 | 23.12 [ 4.08;  130.83] |
| Lahti | 49 | 41.27 [31.22;  54.55] | 19 | 15.91 [10.18;  24.84] | 32 | 26.76 [18.96;  37.78] | 44 | 36.68 [27.33;  49.24] | 43 | 35.89 [26.65;  48.33] | 46 | 38.34 [28.75;  51.13] |
| Myrskylä | 1 | 50.79 [ 8.97;  287.13] | 0 | 0.00 [ 0.00;  193.05] | 1 | 50.79 [ 8.97;  287.13] | 0 | 0.00 [ 0.00;  199.47] | 0 | 0.00 [ 0.00;  203.70] | 0 | 0.00 [ 0.00;  204.90] |
| Orimattila | 9 | 55.13 [29.01;  104.75] | 5 | 30.71 [13.12;  71.89] | 8 | 49.32 [24.99;  97.30] | 7 | 43.50 [21.07;  89.78] | 2 | 12.50 [ 3.43;  45.56] | 8 | 50.37 [25.53;  99.37] |
| Padasjoki | 3 | 95.45 [32.47;  280.27] | 5 | 162.71 [69.52;  380.34] | 3 | 99.11 [33.71;  291.00] | 0 | 0.00 [ 0.00;  129.48] | 1 | 34.53 [ 6.10;  195.34] | 3 | 104.86 [35.67;  307.86] |
| Pukkila | 0 | 0.00 [ 0.00;  194.52] | 0 | 0.00 [ 0.00;  192.86] | 0 | 0.00 [ 0.00;  197.62] | 2 | 105.32 [28.89;  383.21] | 1 | 53.76 [ 9.49;  303.92] | 0 | 0.00 [ 0.00;  209.13] |
| Sysmä | 1 | 24.75 [ 4.37;  140.08] | 0 | 0.00 [ 0.00;  97.08] | 3 | 77.74 [26.44;  228.33] | 1 | 26.65 [ 4.70;  150.78] | 2 | 54.69 [15.00;  199.20] | 2 | 55.08 [15.11;  200.62] |
| **Pirkanmaan** |  |  |  |  |  |  |  |  |  |  |  |  |
| Akaa | 2 | 11.74 [ 3.22;  42.78] | 4 | 23.64 [ 9.19;  60.76] | 2 | 11.93 [ 3.27;  43.48] | 2 | 12.04 [ 3.30;  43.89] | 1 | 6.07 [ 1.07;  34.38] | 2 | 12.20 [ 3.35;  44.48] |
| Hämeenkyrö | 0 | 0.00 [ 0.00;  36.00] | 2 | 18.72 [ 5.14;  68.25] | 0 | 0.00 [ 0.00;  36.23] | 3 | 28.54 [ 9.71;  83.90] | 2 | 19.22 [ 5.27;  70.07] | 4 | 38.67 [15.04;  99.40] |
| Ikaalinen | 1 | 13.88 [ 2.45;  78.56] | 1 | 14.03 [ 2.48;  79.43] | 0 | 0.00 [ 0.00;  53.93] | 0 | 0.00 [ 0.00;  54.82] | 2 | 28.81 [ 7.90;  104.99] | 2 | 29.13 [ 7.99;  106.15] |
| Jämsä | 4 | 18.57 [ 7.22;  47.74] | 6 | 28.22 [12.94;  61.57] | 7 | 33.53 [16.24;  69.20] | 12 | 58.23 [33.32;  101.77] | 13 | 64.41 [37.65;  110.18] | 8 | 40.23 [20.39;  79.37] |
| Juupajoki | 0 | 0.00 [ 0.00;  192.86] | 1 | 51.10 [ 9.02;  288.88] | 1 | 52.52 [ 9.27;  296.91] | 0 | 0.00 [ 0.00;  203.48] | 0 | 0.00 [ 0.00;  207.89] | 0 | 0.00 [ 0.00;  212.96] |
| Kangasala | 21 | 68.61 [44.88;  104.87] | 15 | 48.09 [29.15;  79.34] | 14 | 44.53 [26.53;  74.74] | 11 | 34.73 [19.39;  62.18] | 16 | 50.21 [30.91;  81.55] | 5 | 15.52 [ 6.63;  36.33] |
| Kihniö | 1 | 49.07 [ 8.66;  277.43] | 0 | 0.00 [ 0.00;  192.28] | 1 | 50.84 [ 8.97;  287.42] | 1 | 52.36 [ 9.24;  295.98] | 1 | 53.62 [ 9.47;  303.11] | 0 | 0.00 [ 0.00;  210.39] |
| Kuhmoinen | 3 | 128.53 [43.72;  377.24] | 0 | 0.00 [ 0.00;  167.76] | 3 | 133.21 [45.32;  390.95] | 7 | 312.78 [ 151.59;  644.25] | 6 | 271.99 [ 124.71;  592.15] | 5 | 231.37 [98.87;  540.51] |
| Lempäälä | 9 | 39.94 [21.01;  75.89] | 3 | 13.19 [ 4.49;  38.78] | 9 | 39.42 [20.74;  74.92] | 7 | 30.16 [14.61;  62.26] | 8 | 34.01 [17.23;  67.10] | 9 | 37.77 [19.87;  71.78] |
| Mänttä-Vilppula | 5 | 47.15 [20.14;  110.34] | 2 | 19.14 [ 5.25;  69.77] | 3 | 29.25 [ 9.95;  85.97] | 4 | 40.07 [15.58;  102.99] | 3 | 30.44 [10.35;  89.47] | 5 | 51.69 [22.08;  120.96] |
| Nokia | 8 | 24.12 [12.22;  47.60] | 7 | 21.08 [10.21;  43.51] | 9 | 27.01 [14.21;  51.33] | 10 | 29.83 [16.20;  54.90] | 13 | 38.32 [22.39;  65.55] | 10 | 29.01 [15.76;  53.39] |
| Orivesi | 0 | 0.00 [ 0.00;  40.82] | 1 | 10.74 [ 1.90;  60.81] | 6 | 64.62 [29.62;  140.92] | 2 | 21.69 [ 5.95;  79.06] | 5 | 54.60 [23.32;  127.75] | 0 | 0.00 [ 0.00;  42.63] |
| Pälkäne | 9 | 134.81 [70.94;  256.03] | 6 | 90.54 [41.50;  197.40] | 4 | 60.91 [23.69;  156.52] | 5 | 76.93 [32.87;  179.99] | 6 | 93.24 [42.74;  203.29] | 3 | 46.77 [15.91;  137.42] |
| Parkano | 3 | 44.34 [15.08;  130.29] | 4 | 59.77 [23.25;  153.60] | 0 | 0.00 [ 0.00;  58.51] | 1 | 15.46 [ 2.73;  87.52] | 0 | 0.00 [ 0.00;  59.95] | 2 | 31.49 [ 8.64;  114.74] |
| Pirkkala | 14 | 74.02 [44.10;  124.22] | 11 | 57.40 [32.06;  102.77] | 10 | 51.98 [28.24;  95.67] | 11 | 56.79 [31.72;  101.68] | 9 | 45.86 [24.13;  87.15] | 6 | 30.30 [13.89;  66.09] |
| Ruovesi | 4 | 86.52 [33.65;  222.28] | 0 | 0.00 [ 0.00;  84.08] | 3 | 67.28 [22.88;  197.64] | 5 | 113.69 [48.57;  265.88] | 4 | 93.39 [36.32;  239.90] | 1 | 23.72 [ 4.19;  134.27] |
| Sastamala | 1 | 3.97 [ 0.70;  22.46] | 2 | 7.98 [ 2.19;  29.09] | 5 | 20.15 [ 8.61;  47.15] | 3 | 12.17 [ 4.14;  35.78] | 6 | 24.71 [11.33;  53.92] | 2 | 8.32 [ 2.28;  30.32] |
| Tampere | 40 | 17.77 [13.05;  24.19] | 43 | 18.84 [13.99;  25.37] | 56 | 24.15 [18.60;  31.36] | 71 | 30.18 [23.93;  38.06] | 82 | 34.43 [27.74;  42.73] | 33 | 13.69 [ 9.75;  19.23] |
| Urjala | 0 | 0.00 [ 0.00;  77.89] | 1 | 20.58 [ 3.63;  116.52] | 1 | 20.71 [ 3.66;  117.21] | 0 | 0.00 [ 0.00;  80.10] | 0 | 0.00 [ 0.00;  81.88] | 1 | 21.53 [ 3.80;  121.88] |
| Valkeakoski | 1 | 4.69 [ 0.83;  26.55] | 0 | 0.00 [ 0.00;  17.99] | 3 | 14.19 [ 4.83;  41.73] | 2 | 9.46 [ 2.59;  34.50] | 6 | 28.61 [13.11;  62.41] | 2 | 9.63 [ 2.64;  35.11] |
| Vesilahti | 2 | 44.55 [12.22;  162.31] | 2 | 44.82 [12.29;  163.29] | 1 | 22.42 [ 3.96;  126.90] | 4 | 91.05 [35.41;  233.90] | 0 | 0.00 [ 0.00;  88.13] | 1 | 22.90 [ 4.04;  129.60] |
| Virrat | 1 | 14.28 [ 2.52;  80.86] | 3 | 43.37 [14.75;  127.45] | 2 | 29.22 [ 8.01;  106.50] | 4 | 59.36 [23.08;  152.53] | 4 | 61.12 [23.77;  157.07] | 4 | 61.44 [23.90;  157.89] |
| Ylöjärvi | 4 | 12.22 [ 4.75;  31.41] | 1 | 3.05 [ 0.54;  17.27] | 14 | 42.58 [25.37;  71.47] | 6 | 18.19 [ 8.34;  39.69] | 4 | 12.03 [ 4.68;  30.93] | 4 | 11.99 [ 4.66;  30.84] |
| **Pohjois-Karjalan** |  |  |  |  |  |  |  |  |  |  |  |  |
| Heinävesi | 1 | 27.98 [ 4.94;  158.33] | 1 | 28.46 [ 5.02;  161.03] | 7 | 202.60 [98.18;  417.64] | 2 | 60.08 [16.48;  218.80] | 11 | 338.05 [ 188.87;  604.34] | 2 | 62.58 [17.16;  227.89] |
| Ilomantsi | 2 | 37.48 [10.28;  136.57] | 7 | 133.66 [64.76;  275.67] | 22 | 429.02 [ 283.49;  648.75] | 7 | 140.76 [68.20;  290.29] | 15 | 308.83 [ 187.25;  508.96] | 10 | 210.57 [ 114.42;  387.20] |
| Joensuu | 50 | 66.21 [50.23;  87.27] | 32 | 42.19 [29.89;  59.55] | 54 | 70.99 [54.42;  92.61] | 112 | 146.31 [ 121.62;  176.00] | 119 | 154.85 [ 129.43;  185.25] | 63 | 81.89 [64.01;  104.74] |
| Juuka | 1 | 19.86 [ 3.51;  112.44] | 1 | 20.25 [ 3.57;  114.63] | 5 | 103.80 [44.34;  242.77] | 12 | 254.83 [ 145.84;  444.92] | 15 | 325.66 [ 197.46;  536.65] | 8 | 176.72 [89.57;  348.35] |
| Kitee | 26 | 240.03 [ 163.86;  351.48] | 9 | 83.96 [44.18;  159.51] | 8 | 76.29 [38.66;  150.49] | 20 | 193.09 [ 125.03;  298.07] | 18 | 177.58 [ 112.36;  280.56] | 3 | 30.20 [10.27;  88.77] |
| Kontiolahti | 10 | 67.44 [36.64;  124.12] | 3 | 20.26 [ 6.89;  59.56] | 9 | 60.69 [31.93;  115.31] | 7 | 47.14 [22.84;  97.28] | 8 | 53.98 [27.35;  106.49] | 10 | 67.31 [36.57;  123.87] |
| Lieksa | 7 | 59.46 [28.81;  122.70] | 3 | 25.91 [ 8.81;  76.15] | 14 | 123.93 [73.84;  207.92] | 27 | 243.29 [ 167.26;  353.75] | 41 | 376.70 [ 277.82;  510.60] | 13 | 121.28 [70.89;  207.41] |
| Liperi | 3 | 24.32 [ 8.27;  71.47] | 8 | 65.04 [32.96;  128.29] | 9 | 74.07 [38.98;  140.73] | 14 | 115.27 [68.68;  193.41] | 7 | 57.93 [28.06;  119.53] | 5 | 41.69 [17.81;  97.56] |
| Nurmes | 9 | 87.21 [45.89;  165.67] | 7 | 69.10 [33.48;  142.58] | 10 | 100.17 [54.42;  184.31] | 21 | 214.64 [ 140.43;  327.92] | 36 | 376.88 [ 272.37;  521.30] | 13 | 136.83 [79.98;  233.98] |
| Outokumpu | 8 | 112.06 [56.79;  220.99] | 4 | 56.41 [21.94;  144.96] | 8 | 114.24 [57.90;  225.28] | 6 | 88.20 [40.43;  192.30] | 7 | 104.67 [50.71;  215.91] | 7 | 106.84 [51.76;  220.38] |
| Polvijärvi | 9 | 197.54 [ 103.96;  375.03] | 2 | 44.31 [12.15;  161.42] | 4 | 90.62 [35.25;  232.79] | 6 | 139.31 [63.86;  303.62] | 15 | 353.27 [ 214.21;  582.09] | 7 | 166.63 [80.74;  343.57] |
| Rääkkylä | 1 | 42.57 [ 7.52;  240.76] | 0 | 0.00 [ 0.00;  169.09] | 3 | 133.93 [45.56;  393.04] | 9 | 412.65 [ 217.25;  782.43] | 4 | 188.15 [73.19;  482.79] | 26 | 1,258.47 [ 860.25; 1,837.61] |
| Tohmajärvi | 4 | 84.42 [32.84;  216.89] | 5 | 107.46 [45.91;  251.32] | 5 | 109.39 [46.73;  255.82] | 6 | 133.87 [61.37;  291.78] | 6 | 137.58 [63.07;  299.86] | 3 | 69.65 [23.69;  204.60] |
| **Pohjois-Pohjanmaan** | |  |  |  |  |  |  |  |  |  |  |  |
| Alavieska | 0 | 0.00 [ 0.00;  142.76] | 0 | 0.00 [ 0.00;  145.35] | 0 | 0.00 [ 0.00;  146.97] | 0 | 0.00 [ 0.00;  149.08] | 0 | 0.00 [ 0.00;  152.27] | 2 | 79.46 [21.79;  289.27] |
| Haapajärvi | 1 | 13.44 [ 2.37;  76.12] | 0 | 0.00 [ 0.00;  52.37] | 0 | 0.00 [ 0.00;  52.95] | 1 | 13.99 [ 2.47;  79.22] | 1 | 14.27 [ 2.52;  80.77] | 1 | 14.50 [ 2.56;  82.10] |
| Haapavesi | 5 | 69.76 [29.80;  163.22] | 0 | 0.00 [ 0.00;  54.09] | 0 | 0.00 [ 0.00;  55.08] | 0 | 0.00 [ 0.00;  56.02] | 1 | 14.80 [ 2.61;  83.78] | 2 | 30.00 [ 8.23;  109.32] |
| Hailuoto | 0 | 0.00 [ 0.00;  385.36] | 1 | 100.60 [17.76;  567.65] | 1 | 103.41 [18.26;  583.43] | 2 | 205.34 [56.33;  745.59] | 4 | 417.10 [ 162.32; 1,067.53] | 0 | 0.00 [ 0.00;  403.16] |
| Ii | 0 | 0.00 [ 0.00;  39.74] | 1 | 10.39 [ 1.83;  58.81] | 5 | 50.17 [21.43;  117.40] | 3 | 30.42 [10.35;  89.41] | 5 | 50.79 [21.70;  118.86] | 6 | 60.93 [27.93;  132.87] |
| Kalajoki | 5 | 39.62 [16.92;  92.71] | 2 | 15.89 [ 4.36;  57.93] | 1 | 7.99 [ 1.41;  45.25] | 2 | 16.15 [ 4.43;  58.86] | 6 | 48.49 [22.23;  105.77] | 3 | 24.19 [ 8.23;  71.11] |
| Kärsämäki | 0 | 0.00 [ 0.00;  144.32] | 0 | 0.00 [ 0.00;  144.48] | 1 | 38.30 [ 6.76;  216.64] | 0 | 0.00 [ 0.00;  146.80] | 0 | 0.00 [ 0.00;  148.90] | 3 | 118.20 [40.21;  346.97] |
| Kempele | 2 | 11.72 [ 3.21;  42.72] | 0 | 0.00 [ 0.00;  22.20] | 3 | 17.11 [ 5.82;  50.29] | 1 | 5.58 [ 0.98;  31.60] | 4 | 21.79 [ 8.47;  56.03] | 4 | 21.28 [ 8.28;  54.71] |
| Kuusamo | 1 | 6.37 [ 1.13;  36.10] | 2 | 12.88 [ 3.53;  46.94] | 0 | 0.00 [ 0.00;  24.96] | 0 | 0.00 [ 0.00;  25.25] | 5 | 33.04 [14.11;  77.32] | 0 | 0.00 [ 0.00;  25.24] |
| Liminka | 1 | 10.06 [ 1.78;  56.99] | 0 | 0.00 [ 0.00;  38.40] | 3 | 29.61 [10.07;  87.02] | 2 | 19.68 [ 5.40;  71.74] | 5 | 49.06 [20.96;  114.81] | 0 | 0.00 [ 0.00;  37.51] |
| Lumijoki | 3 | 144.51 [49.16;  424.03] | 0 | 0.00 [ 0.00;  182.16] | 1 | 48.05 [ 8.48;  271.70] | 0 | 0.00 [ 0.00;  186.86] | 3 | 148.51 [50.52;  435.76] | 3 | 147.35 [50.12;  432.34] |
| Merijärvi | 0 | 0.00 [ 0.00;  337.61] | 0 | 0.00 [ 0.00;  338.50] | 0 | 0.00 [ 0.00;  342.12] | 0 | 0.00 [ 0.00;  346.75] | 0 | 0.00 [ 0.00;  351.51] | 0 | 0.00 [ 0.00;  355.09] |
| Muhos | 0 | 0.00 [ 0.00;  42.37] | 0 | 0.00 [ 0.00;  42.69] | 0 | 0.00 [ 0.00;  42.57] | 0 | 0.00 [ 0.00;  42.76] | 0 | 0.00 [ 0.00;  43.11] | 0 | 0.00 [ 0.00;  43.13] |
| Nivala | 4 | 36.78 [14.30;  94.54] | 1 | 9.18 [ 1.62;  52.01] | 1 | 9.25 [ 1.63;  52.36] | 1 | 9.31 [ 1.64;  52.74] | 0 | 0.00 [ 0.00;  36.09] | 4 | 38.10 [14.82;  97.92] |
| Oulainen | 0 | 0.00 [ 0.00;  50.45] | 0 | 0.00 [ 0.00;  51.10] | 1 | 13.38 [ 2.36;  75.78] | 0 | 0.00 [ 0.00;  51.68] | 0 | 0.00 [ 0.00;  52.68] | 0 | 0.00 [ 0.00;  53.66] |
| Oulu | 12 | 6.04 [ 3.46;  10.57] | 11 | 5.49 [ 3.06;  9.82] | 18 | 8.92 [ 5.64;  14.10] | 14 | 6.88 [ 4.10;  11.54] | 110 | 53.53 [44.42;  64.51] | 68 | 32.80 [25.88;  41.57] |
| Pudasjärvi | 0 | 0.00 [ 0.00;  46.50] | 0 | 0.00 [ 0.00;  46.90] | 1 | 12.34 [ 2.18;  69.88] | 0 | 0.00 [ 0.00;  48.06] | 1 | 12.70 [ 2.24;  71.92] | 1 | 12.86 [ 2.27;  72.79] |
| Pyhäjärvi | 0 | 0.00 [ 0.00;  69.73] | 2 | 36.72 [10.07;  133.81] | 1 | 18.74 [ 3.31;  106.07] | 0 | 0.00 [ 0.00;  73.14] | 3 | 58.47 [19.89;  171.77] | 0 | 0.00 [ 0.00;  76.27] |
| Pyhäjoki | 0 | 0.00 [ 0.00;  119.49] | 0 | 0.00 [ 0.00;  120.35] | 2 | 62.72 [17.20;  228.39] | 2 | 63.57 [17.44;  231.51] | 1 | 32.50 [ 5.74;  183.87] | 3 | 98.33 [33.45;  288.71] |
| Pyhäntä | 0 | 0.00 [ 0.00;  241.47] | 0 | 0.00 [ 0.00;  242.69] | 0 | 0.00 [ 0.00;  242.69] | 0 | 0.00 [ 0.00;  246.11] | 0 | 0.00 [ 0.00;  242.85] | 0 | 0.00 [ 0.00;  240.57] |
| Raahe | 4 | 15.90 [ 6.18;  40.87] | 4 | 15.99 [ 6.22;  41.12] | 7 | 28.00 [13.56;  57.79] | 13 | 52.40 [30.62;  89.63] | 29 | 117.51 [81.83;  168.71] | 16 | 65.70 [40.45;  106.70] |
| Sievi | 0 | 0.00 [ 0.00;  74.91] | 1 | 19.73 [ 3.48;  111.67] | 0 | 0.00 [ 0.00;  76.24] | 1 | 20.08 [ 3.54;  113.66] | 0 | 0.00 [ 0.00;  78.18] | 0 | 0.00 [ 0.00;  79.40] |
| Siikajoki | 0 | 0.00 [ 0.00;  70.23] | 1 | 18.64 [ 3.29;  105.49] | 0 | 0.00 [ 0.00;  71.85] | 2 | 38.10 [10.45;  138.83] | 5 | 97.18 [41.52;  227.31] | 1 | 19.86 [ 3.51;  112.44] |
| Siikalatva | 0 | 0.00 [ 0.00;  67.62] | 0 | 0.00 [ 0.00;  68.76] | 0 | 0.00 [ 0.00;  70.47] | 4 | 75.46 [29.35;  193.87] | 0 | 0.00 [ 0.00;  73.38] | 1 | 19.22 [ 3.39;  108.80] |
| Taivalkoski | 0 | 0.00 [ 0.00;  91.40] | 0 | 0.00 [ 0.00;  92.86] | 1 | 24.64 [ 4.35;  139.46] | 1 | 24.85 [ 4.39;  140.64] | 1 | 25.15 [ 4.44;  142.34] | 0 | 0.00 [ 0.00;  98.00] |
| Tyrnävä | 0 | 0.00 [ 0.00;  56.52] | 2 | 29.63 [ 8.13;  107.98] | 3 | 44.58 [15.16;  130.99] | 2 | 29.59 [ 8.12;  107.85] | 4 | 60.27 [23.44;  154.87] | 1 | 15.14 [ 2.67;  85.74] |
| Utajärvi | 0 | 0.00 [ 0.00;  134.09] | 0 | 0.00 [ 0.00;  135.84] | 0 | 0.00 [ 0.00;  138.59] | 0 | 0.00 [ 0.00;  141.97] | 0 | 0.00 [ 0.00;  143.35] | 0 | 0.00 [ 0.00;  146.46] |
| Vaala | 0 | 0.00 [ 0.00;  124.81] | 0 | 0.00 [ 0.00;  126.20] | 0 | 0.00 [ 0.00;  130.45] | 0 | 0.00 [ 0.00;  133.72] | 0 | 0.00 [ 0.00;  137.40] | 1 | 36.54 [ 6.45;  206.68] |
| Ylivieska | 2 | 13.30 [ 3.65;  48.48] | 1 | 6.58 [ 1.16;  37.26] | 0 | 0.00 [ 0.00;  25.18] | 0 | 0.00 [ 0.00;  25.25] | 1 | 6.56 [ 1.16;  37.13] | 0 | 0.00 [ 0.00;  25.09] |
| **Pohjois-Savon** |  |  |  |  |  |  |  |  |  |  |  |  |
| Iisalmi | 4 | 18.23 [ 7.09;  46.86] | 2 | 9.19 [ 2.52;  33.50] | 8 | 36.97 [18.73;  72.94] | 13 | 60.54 [35.39;  103.57] | 27 | 126.36 [86.86;  183.78] | 7 | 33.14 [16.05;  68.39] |
| Kaavi | 1 | 31.31 [ 5.53;  177.14] | 1 | 31.71 [ 5.60;  179.39] | 0 | 0.00 [ 0.00;  125.87] | 1 | 33.44 [ 5.90;  189.21] | 4 | 138.26 [53.78;  354.99] | 0 | 0.00 [ 0.00;  136.67] |
| Keitele | 3 | 126.10 [42.90;  370.12] | 0 | 0.00 [ 0.00;  163.48] | 0 | 0.00 [ 0.00;  166.09] | 3 | 133.69 [45.48;  392.34] | 4 | 181.65 [70.66;  466.16] | 1 | 46.40 [ 8.19;  262.39] |
| Kiuruvesi | 2 | 23.26 [ 6.38;  84.76] | 2 | 23.69 [ 6.50;  86.33] | 3 | 36.22 [12.32;  106.44] | 4 | 49.06 [19.08;  126.09] | 6 | 75.02 [34.39;  163.59] | 1 | 12.73 [ 2.25;  72.09] |
| Kuopio | 114 | 97.50 [81.18;  117.11] | 77 | 65.40 [52.33;  81.72] | 88 | 74.44 [60.43;  91.70] | 156 | 131.46 [ 112.40;  153.76] | 131 | 109.82 [92.57;  130.29] | 55 | 45.75 [35.16;  59.54] |
| Lapinlahti | 10 | 100.18 [54.43;  184.33] | 15 | 151.79 [92.01;  250.31] | 17 | 175.40 [ 109.55;  280.74] | 23 | 239.16 [ 159.42;  358.63] | 21 | 221.40 [ 144.86;  338.25] | 19 | 203.03 [ 130.02;  316.91] |
| Leppävirta | 4 | 40.19 [15.63;  103.30] | 3 | 30.41 [10.34;  89.38] | 2 | 20.45 [ 5.61;  74.52] | 3 | 31.09 [10.57;  91.37] | 3 | 31.73 [10.79;  93.26] | 3 | 31.91 [10.85;  93.78] |
| Pielavesi | 10 | 210.97 [ 114.64;  387.94] | 10 | 212.90 [ 115.69;  391.49] | 14 | 302.77 [ 180.44;  507.60] | 22 | 489.11 [ 323.23;  739.48] | 24 | 546.57 [ 367.58;  812.02] | 6 | 138.86 [63.65;  302.64] |
| Rautalampi | 3 | 90.83 [30.89;  266.72] | 1 | 30.41 [ 5.37;  172.08] | 1 | 30.72 [ 5.42;  173.83] | 5 | 156.45 [66.84;  365.72] | 1 | 32.04 [ 5.66;  181.28] | 1 | 32.75 [ 5.78;  185.31] |
| Rautavaara | 0 | 0.00 [ 0.00;  220.67] | 0 | 0.00 [ 0.00;  222.46] | 0 | 0.00 [ 0.00;  225.72] | 0 | 0.00 [ 0.00;  232.13] | 1 | 62.42 [11.02;  352.74] | 0 | 0.00 [ 0.00;  245.49] |
| Siilinjärvi | 18 | 82.59 [52.25;  130.53] | 19 | 87.28 [55.89;  136.29] | 29 | 133.91 [93.25;  192.24] | 43 | 198.39 [ 147.33;  267.11] | 23 | 107.36 [71.55;  161.06] | 5 | 23.53 [10.05;  55.07] |
| Sonkajärvi | 0 | 0.00 [ 0.00;  89.72] | 0 | 0.00 [ 0.00;  91.40] | 0 | 0.00 [ 0.00;  94.18] | 3 | 75.62 [25.72;  222.12] | 3 | 76.98 [26.18;  226.11] | 1 | 26.03 [ 4.60;  147.33] |
| Suonenjoki | 9 | 121.79 [64.09;  231.31] | 1 | 13.68 [ 2.41;  77.43] | 6 | 82.58 [37.85;  180.06] | 7 | 97.97 [47.47;  202.11] | 2 | 28.31 [ 7.76;  103.18] | 2 | 28.86 [ 7.91;  105.16] |
| Tervo | 9 | 559.70 [ 294.74; 1,060.32] | 5 | 310.37 [ 132.64;  724.50] | 3 | 189.27 [64.39;  555.02] | 4 | 255.26 [99.31;  654.52] | 7 | 460.53 [ 223.26;  947.56] | 0 | 0.00 [ 0.00;  254.93] |
| Tuusniemi | 4 | 147.11 [57.22;  377.67] | 3 | 113.51 [38.61;  333.21] | 1 | 38.51 [ 6.80;  217.80] | 1 | 39.20 [ 6.92;  221.72] | 3 | 121.11 [41.20;  355.50] | 1 | 41.10 [ 7.26;  232.46] |
| Varkaus | 37 | 171.00 [ 124.09;  235.59] | 22 | 102.48 [67.69;  155.12] | 20 | 94.54 [61.21;  145.99] | 20 | 96.02 [62.17;  148.27] | 36 | 175.90 [ 127.09;  243.41] | 17 | 83.83 [52.35;  134.23] |
| Vesanto | 2 | 91.28 [25.04;  332.23] | 3 | 139.66 [47.51;  409.84] | 0 | 0.00 [ 0.00;  183.11] | 1 | 48.59 [ 8.58;  274.73] | 0 | 0.00 [ 0.00;  190.37] | 2 | 101.42 [27.82;  369.05] |
| Vieremä | 0 | 0.00 [ 0.00;  102.14] | 0 | 0.00 [ 0.00;  103.13] | 0 | 0.00 [ 0.00;  104.14] | 0 | 0.00 [ 0.00;  104.39] | 0 | 0.00 [ 0.00;  107.22] | 1 | 28.39 [ 5.01;  160.66] |
| **Satakunnan** |  |  |  |  |  |  |  |  |  |  |  |  |
| Eura | 2 | 16.49 [ 4.52;  60.11] | 2 | 16.66 [ 4.57;  60.73] | 3 | 25.19 [ 8.57;  74.04] | 5 | 42.56 [18.18;  99.60] | 7 | 60.18 [29.15;  124.18] | 5 | 43.54 [18.60;  101.90] |
| Eurajoki | 7 | 75.37 [36.52;  155.52] | 2 | 21.24 [ 5.82;  77.40] | 7 | 73.52 [35.62;  151.70] | 3 | 31.73 [10.79;  93.26] | 6 | 63.82 [29.25;  139.17] | 7 | 74.06 [35.88;  152.80] |
| Harjavalta | 5 | 68.53 [29.28;  160.34] | 3 | 41.44 [14.09;  121.77] | 2 | 27.97 [ 7.67;  101.93] | 0 | 0.00 [ 0.00;  54.71] | 0 | 0.00 [ 0.00;  55.39] | 1 | 14.56 [ 2.57;  82.42] |
| Huittinen | 2 | 19.10 [ 5.24;  69.61] | 1 | 9.61 [ 1.70;  54.43] | 1 | 9.80 [ 1.73;  55.48] | 3 | 29.73 [10.11;  87.38] | 0 | 0.00 [ 0.00;  38.23] | 0 | 0.00 [ 0.00;  38.64] |
| Jämijärvi | 0 | 0.00 [ 0.00;  196.81] | 1 | 52.22 [ 9.22;  295.21] | 1 | 53.56 [ 9.46;  302.78] | 0 | 0.00 [ 0.00;  211.90] | 0 | 0.00 [ 0.00;  220.41] | 1 | 58.58 [10.34;  331.10] |
| Kankaanpää | 4 | 29.49 [11.47;  75.82] | 3 | 22.39 [ 7.62;  65.83] | 5 | 37.62 [16.07;  88.04] | 5 | 38.06 [16.26;  89.07] | 5 | 38.74 [16.55;  90.67] | 3 | 23.51 [ 8.00;  69.12] |
| Karvia | 0 | 0.00 [ 0.00;  154.97] | 1 | 40.83 [ 7.21;  230.94] | 0 | 0.00 [ 0.00;  159.61] | 1 | 41.84 [ 7.39;  236.63] | 1 | 42.70 [ 7.54;  241.48] | 0 | 0.00 [ 0.00;  165.16] |
| Kokemäki | 2 | 26.35 [ 7.23;  96.02] | 1 | 13.34 [ 2.35;  75.51] | 0 | 0.00 [ 0.00;  52.02] | 2 | 27.68 [ 7.59;  100.87] | 1 | 14.08 [ 2.49;  79.71] | 1 | 14.26 [ 2.52;  80.73] |
| Merikarvia | 1 | 31.40 [ 5.54;  177.64] | 0 | 0.00 [ 0.00;  121.07] | 0 | 0.00 [ 0.00;  121.57] | 1 | 32.10 [ 5.67;  181.63] | 1 | 32.61 [ 5.76;  184.47] | 3 | 97.85 [33.28;  287.30] |
| Nakkila | 0 | 0.00 [ 0.00;  67.93] | 2 | 36.05 [ 9.89;  131.35] | 1 | 18.11 [ 3.20;  102.53] | 6 | 110.35 [50.59;  240.57] | 3 | 56.30 [19.15;  165.40] | 2 | 38.05 [10.44;  138.65] |
| Pomarkku | 2 | 89.29 [24.49;  324.98] | 2 | 89.57 [24.57;  325.99] | 2 | 92.34 [25.33;  336.06] | 1 | 46.60 [ 8.23;  263.49] | 1 | 47.87 [ 8.45;  270.67] | 0 | 0.00 [ 0.00;  185.86] |
| Pori | 15 | 17.57 [10.65;  28.99] | 16 | 18.81 [11.58;  30.56] | 30 | 35.47 [24.85;  50.63] | 27 | 31.99 [21.99;  46.54] | 37 | 44.08 [31.99;  60.75] | 55 | 65.72 [50.50;  85.53] |
| Rauma | 41 | 102.99 [75.93;  139.68] | 43 | 108.55 [80.60;  146.17] | 50 | 126.20 [95.75;  166.32] | 68 | 172.76 [ 136.31;  218.94] | 55 | 140.29 [ 107.80;  182.54] | 71 | 181.86 [ 144.22;  229.31] |
| Säkylä | 1 | 14.14 [ 2.50;  80.08] | 0 | 0.00 [ 0.00;  54.94] | 2 | 28.97 [ 7.95;  105.59] | 3 | 44.05 [14.98;  129.43] | 2 | 29.76 [ 8.16;  108.44] | 1 | 15.05 [ 2.66;  85.19] |
| Siikainen | 0 | 0.00 [ 0.00;  250.94] | 0 | 0.00 [ 0.00;  256.47] | 1 | 67.75 [11.96;  382.77] | 1 | 68.59 [12.11;  387.49] | 0 | 0.00 [ 0.00;  266.61] | 0 | 0.00 [ 0.00;  276.59] |
| Ulvila | 3 | 22.47 [ 7.64;  66.04] | 7 | 52.58 [25.47;  108.51] | 7 | 52.88 [25.62;  109.13] | 3 | 23.04 [ 7.84;  67.72] | 3 | 23.31 [ 7.93;  68.51] | 5 | 39.26 [16.77;  91.88] |
| **Tuntematon** |  |  |  |  |  |  |  |  |  |  |  |  |
| Puuttuu | 0 | NA [ NA;  NA] | 0 | NA [ NA;  NA] | 0 | NA [ NA;  NA] | 0 | NA [ NA;  NA] | 0 | NA [ NA;  NA] | 4 | NA [ NA;  NA] |
| Tuntematon | 0 | NA [ NA;  NA] | 0 | NA [ NA;  NA] | 1 | NA [ NA;  NA] | 0 | NA [ NA;  NA] | 1 | NA [ NA;  NA] | 2 | NA [ NA;  NA] |
| Ulkomaat | 39 | NA [ NA;  NA] | 36 | NA [ NA;  NA] | 47 | NA [ NA;  NA] | 38 | NA [ NA;  NA] | 59 | NA [ NA;  NA] | 25 | NA [ NA;  NA] |
| Ulkomailla | 1 | NA [ NA;  NA] | 2 | NA [ NA;  NA] | 0 | NA [ NA;  NA] | 0 | NA [ NA;  NA] | 0 | NA [ NA;  NA] | 1 | NA [ NA;  NA] |
| Virheellinen | 1 | NA [ NA;  NA] | 1 | NA [ NA;  NA] | 0 | NA [ NA;  NA] | 0 | NA [ NA;  NA] | 0 | NA [ NA;  NA] | 44 | NA [ NA;  NA] |
| Virheellinen kuntakoodi | 0 | NA [ NA;  NA] | 0 | NA [ NA;  NA] | 0 | NA [ NA;  NA] | 0 | NA [ NA;  NA] | 0 | NA [ NA;  NA] | 0 | NA [ NA;  NA] |
| **Vaasan** |  |  |  |  |  |  |  |  |  |  |  |  |
| Kaskinen | 1 | 77.82 [13.74;  439.49] | 0 | 0.00 [ 0.00;  295.53] | 1 | 78.49 [13.86;  443.28] | 1 | 79.24 [13.99;  447.48] | 1 | 80.26 [14.17;  453.21] | 1 | 78.25 [13.81;  441.89] |
| Korsnäs | 0 | 0.00 [ 0.00;  174.23] | 1 | 46.06 [ 8.13;  260.46] | 1 | 46.43 [ 8.20;  262.51] | 1 | 47.13 [ 8.32;  266.46] | 0 | 0.00 [ 0.00;  184.61] | 1 | 48.36 [ 8.54;  273.41] |
| Kristiinankaupunki | 1 | 14.72 [ 2.60;  83.35] | 1 | 14.87 [ 2.62;  84.16] | 2 | 30.13 [ 8.26;  109.80] | 3 | 45.48 [15.47;  133.65] | 2 | 30.84 [ 8.46;  112.37] | 1 | 15.62 [ 2.76;  88.40] |
| Laihia | 2 | 24.72 [ 6.78;  90.10] | 2 | 24.57 [ 6.74;  89.56] | 1 | 12.42 [ 2.19;  70.33] | 4 | 49.64 [19.31;  127.58] | 7 | 87.31 [42.30;  180.14] | 1 | 12.51 [ 2.21;  70.81] |
| Luoto | 8 | 155.43 [78.78;  306.43] | 15 | 289.80 [ 175.71;  477.62] | 14 | 265.96 [ 158.50;  445.95] | 8 | 149.81 [75.93;  295.36] | 7 | 129.22 [62.61;  266.52] | 7 | 126.49 [61.29;  260.89] |
| Maalahti | 8 | 144.27 [73.12;  284.45] | 6 | 108.75 [49.85;  237.09] | 6 | 109.55 [50.22;  238.82] | 6 | 109.55 [50.22;  238.82] | 6 | 109.59 [50.24;  238.90] | 2 | 36.69 [10.06;  133.69] |
| Mustasaari | 3 | 15.54 [ 5.29;  45.69] | 6 | 30.96 [14.19;  67.54] | 6 | 30.95 [14.19;  67.52] | 15 | 77.14 [46.76;  127.25] | 6 | 30.85 [14.14;  67.30] | 3 | 15.42 [ 5.24;  45.34] |
| Närpiö | 3 | 31.96 [10.87;  93.93] | 3 | 31.78 [10.81;  93.41] | 2 | 21.04 [ 5.77;  76.68] | 4 | 42.23 [16.43;  108.55] | 2 | 21.10 [ 5.79;  76.90] | 1 | 10.46 [ 1.85;  59.24] |
| Pedersören kunta | 5 | 44.93 [19.19;  105.14] | 3 | 27.11 [ 9.22;  79.68] | 4 | 36.09 [14.03;  92.76] | 9 | 81.70 [42.99;  155.21] | 6 | 54.15 [24.82;  118.09] | 7 | 62.65 [30.35;  129.27] |
| Pietarsaari | 34 | 174.93 [ 125.22;  244.34] | 32 | 165.14 [ 117.01;  233.03] | 28 | 144.49 [99.99;  208.75] | 30 | 155.62 [ 109.03;  222.06] | 24 | 124.95 [83.98;  185.86] | 16 | 83.92 [51.66;  136.28] |
| Uusikaarlepyy | 18 | 237.97 [ 150.58;  375.87] | 19 | 252.79 [ 161.90;  394.51] | 16 | 212.74 [ 130.99;  345.31] | 16 | 214.62 [ 132.15;  348.37] | 15 | 200.96 [ 121.83;  331.33] | 16 | 213.93 [ 131.73;  347.25] |
| Vaasa | 51 | 75.42 [57.37;  99.14] | 26 | 38.45 [26.24;  56.33] | 40 | 59.35 [43.59;  80.81] | 58 | 85.86 [66.43;  110.97] | 48 | 70.97 [53.54;  94.07] | 18 | 26.65 [16.86;  42.12] |
| Vöyri | 3 | 44.68 [15.20;  131.30] | 1 | 14.96 [ 2.64;  84.70] | 0 | 0.00 [ 0.00;  58.03] | 1 | 15.12 [ 2.67;  85.61] | 1 | 15.48 [ 2.73;  87.63] | 0 | 0.00 [ 0.00;  60.10] |
| **Varsinais-Suomen** |  |  |  |  |  |  |  |  |  |  |  |  |
| Aura | 2 | 50.18 [13.76;  182.77] | 2 | 50.20 [13.77;  182.87] | 3 | 75.17 [25.57;  220.79] | 0 | 0.00 [ 0.00;  96.33] | 5 | 126.87 [54.20;  296.67] | 2 | 50.52 [13.85;  184.02] |
| Kaarina | 66 | 202.52 [ 159.23;  257.54] | 60 | 183.27 [ 142.42;  235.81] | 89 | 268.89 [ 218.58;  330.74] | 78 | 233.13 [ 186.85;  290.83] | 79 | 232.78 [ 186.84;  289.99] | 123 | 354.80 [ 297.48;  423.13] |
| Kemiönsaari | 23 | 332.90 [ 221.94;  499.06] | 14 | 203.73 [ 121.40;  341.69] | 18 | 264.98 [ 167.68;  418.49] | 15 | 223.08 [ 135.24;  367.77] | 28 | 421.69 [ 291.92;  608.79] | 17 | 257.22 [ 160.67;  411.58] |
| Koski Tl | 0 | 0.00 [ 0.00;  159.87] | 1 | 41.39 [ 7.31;  234.09] | 1 | 42.39 [ 7.48;  239.74] | 4 | 170.94 [66.49;  438.72] | 0 | 0.00 [ 0.00;  166.16] | 2 | 87.26 [23.93;  317.62] |
| Kustavi | 17 | 1,899.44 [1,189.25; 3,020.78] | 9 | 991.19 [ 522.33;  1,872.98] | 18 | 1,950.16 [1,237.08;  3,061.55] | 10 | 1,079.91 [ 587.63; 1,976.40] | 17 | 1,791.36 [1,121.40; 2,850.03] | 21 | 2,182.95 [1,432.15; 3,314.12] |
| Laitila | 3 | 35.21 [11.98;  103.48] | 7 | 82.16 [39.80;  169.51] | 11 | 127.76 [71.36;  228.64] | 8 | 92.52 [46.89;  182.47] | 3 | 34.93 [11.88;  102.66] | 13 | 153.52 [89.74;  262.50] |
| Länsi-Turunmaa | 122 | 789.29 [ 661.49;  941.53] | 54 | 350.69 [ 268.90;  457.26] | 100 | 654.24 [ 538.24;  795.03] | 38 | 249.72 [ 182.00;  342.55] | 35 | 231.30 [ 166.36;  321.49] | 39 | 258.19 [ 188.94;  352.74] |
| Lieto | 24 | 124.59 [83.74;  185.33] | 7 | 36.05 [17.46;  74.40] | 16 | 81.65 [50.27;  132.60] | 12 | 60.51 [34.62;  105.75] | 7 | 35.01 [16.96;  72.26] | 24 | 119.13 [80.07;  177.21] |
| Loimaa | 4 | 24.29 [ 9.45;  62.45] | 2 | 12.29 [ 3.37;  44.82] | 2 | 12.38 [ 3.40;  45.15] | 6 | 37.43 [17.15;  81.63] | 0 | 0.00 [ 0.00;  24.19] | 1 | 6.34 [ 1.12;  35.91] |
| Marttila | 3 | 147.93 [50.32;  434.04] | 0 | 0.00 [ 0.00;  189.72] | 1 | 50.30 [ 8.88;  284.39] | 1 | 49.55 [ 8.75;  280.17] | 2 | 99.35 [27.25;  361.55] | 1 | 50.03 [ 8.83;  282.83] |
| Masku | 13 | 133.94 [78.29;  229.04] | 10 | 103.36 [56.15;  190.17] | 13 | 134.63 [78.70;  230.22] | 13 | 136.07 [79.54;  232.68] | 14 | 146.84 [87.49;  246.35] | 17 | 178.14 [ 111.26;  285.12] |
| Mynämäki | 6 | 76.35 [34.99;  166.48] | 6 | 76.51 [35.07;  166.84] | 10 | 127.58 [69.32;  234.71] | 13 | 167.40 [97.86;  286.21] | 12 | 156.78 [89.71;  273.86] | 13 | 170.05 [99.41;  290.74] |
| Naantali | 44 | 232.06 [ 172.92;  311.35] | 47 | 246.49 [ 185.42;  327.59] | 59 | 307.82 [ 238.73;  396.82] | 35 | 181.87 [ 130.80;  252.81] | 71 | 367.61 [ 291.57;  463.39] | 70 | 360.32 [ 285.32;  454.96] |
| Nousiainen | 6 | 123.48 [56.60;  269.16] | 7 | 145.38 [70.44;  299.80] | 5 | 103.89 [44.38;  242.97] | 5 | 105.64 [45.13;  247.08] | 7 | 148.46 [71.93;  306.16] | 10 | 213.08 [ 115.79;  391.82] |
| Oripää | 1 | 72.62 [12.82;  410.22] | 0 | 0.00 [ 0.00;  281.05] | 0 | 0.00 [ 0.00;  277.19] | 0 | 0.00 [ 0.00;  280.84] | 0 | 0.00 [ 0.00;  288.22] | 0 | 0.00 [ 0.00;  287.14] |
| Paimio | 8 | 75.33 [38.18;  148.59] | 11 | 102.68 [57.35;  183.78] | 16 | 149.11 [91.81;  242.10] | 15 | 138.48 [83.94;  228.37] | 16 | 147.47 [90.79;  239.43] | 23 | 210.58 [ 140.37;  315.81] |
| Pöytyä | 3 | 35.04 [11.92;  102.97] | 6 | 70.56 [32.34;  153.88] | 4 | 47.49 [18.47;  122.07] | 2 | 24.00 [ 6.58;  87.48] | 2 | 24.17 [ 6.63;  88.08] | 12 | 145.83 [83.44;  254.74] |
| Punkalaidun | 3 | 98.39 [33.47;  288.90] | 0 | 0.00 [ 0.00;  127.76] | 0 | 0.00 [ 0.00;  130.09] | 2 | 69.06 [18.94;  251.47] | 0 | 0.00 [ 0.00;  135.65] | 0 | 0.00 [ 0.00;  137.74] |
| Pyhäranta | 5 | 234.08 [ 100.03;  546.82] | 10 | 481.93 [ 261.99;  884.88] | 9 | 433.32 [ 228.14;  821.51] | 6 | 295.86 [ 135.66;  644.00] | 10 | 499.00 [ 271.28;  916.14] | 11 | 551.65 [ 308.32;  985.15] |
| Raisio | 49 | 201.73 [ 152.63;  266.57] | 31 | 127.66 [89.96;  181.14] | 59 | 243.46 [ 188.80;  313.89] | 23 | 95.13 [63.40;  142.71] | 51 | 212.01 [ 161.30;  278.61] | 57 | 233.54 [ 180.31;  302.43] |
| Rusko | 14 | 229.13 [ 136.54;  384.27] | 8 | 130.36 [66.07;  257.04] | 14 | 223.54 [ 133.21;  374.89] | 11 | 175.97 [98.29;  314.85] | 7 | 110.64 [53.60;  228.21] | 9 | 141.64 [74.54;  269.00] |
| Salo | 13 | 24.12 [14.10;  41.27] | 14 | 26.15 [15.58;  43.89] | 31 | 58.51 [41.22;  83.03] | 24 | 45.87 [30.83;  68.25] | 33 | 63.67 [45.34;  89.39] | 24 | 46.55 [31.28;  69.25] |
| Sauvo | 5 | 165.62 [70.76;  387.13] | 4 | 131.28 [51.06;  337.08] | 1 | 33.26 [ 5.87;  188.14] | 2 | 66.80 [18.32;  243.25] | 6 | 203.74 [93.41;  443.80] | 4 | 135.59 [52.74;  348.14] |
| Somero | 6 | 65.98 [30.24;  143.90] | 3 | 33.23 [11.30;  97.67] | 4 | 44.85 [17.44;  115.27] | 0 | 0.00 [ 0.00;  43.50] | 3 | 34.44 [11.71;  101.21] | 6 | 69.40 [31.81;  151.33] |
| Taivassalo | 2 | 122.47 [33.59;  445.47] | 9 | 554.87 [ 292.19;  1,051.20] | 10 | 604.59 [ 328.74;  1,109.37] | 9 | 541.52 [ 285.15; 1,025.98] | 9 | 549.12 [ 289.16; 1,040.33] | 8 | 482.22 [ 244.55;  948.68] |
| Turku | 272 | 146.31 [ 129.93;  164.74] | 317 | 168.97 [ 151.38;  188.61] | 331 | 174.51 [ 156.71;  194.33] | 321 | 167.77 [ 150.41;  187.14] | 298 | 154.43 [ 137.88;  172.98] | 247 | 127.06 [ 112.18;  143.92] |
| Uusikaupunki | 50 | 322.37 [ 244.63;  424.72] | 31 | 201.25 [ 141.82;  285.51] | 64 | 406.30 [ 318.33;  518.45] | 51 | 324.84 [ 247.17;  426.81] | 60 | 386.55 [ 300.45;  497.20] | 71 | 461.70 [ 366.23;  581.91] |
| Vehmaa | 1 | 43.94 [ 7.76;  248.47] | 1 | 43.92 [ 7.75;  248.36] | 0 | 0.00 [ 0.00;  165.59] | 3 | 131.29 [44.66;  385.31] | 1 | 43.61 [ 7.70;  246.63] | 3 | 130.89 [44.52;  384.14] |
| **Total Finland** | 5,512 | 100.45 [97.83;  103.14] | 4,917 | 89.35 [86.88;  91.88] | 5,669 | 102.83 [ 100.19;  105.54] | 5,896 | 106.85 [ 104.16;  109.61] | 6,006 | 108.70 [ 105.99;  111.48] | 5,185 | 93.70 [91.18;  96.28] |
