# Supplementary material for: Incidence of Lyme Borreliosis in Finland: Exploring Observed Trends Over Time Using Public Surveillance Data, 2015–2020
Source: Vector Borne Zoonotic Dis. 2023 Apr 12;23(4):256–64. doi: 10.1089/vbz.2022.0047 (PMC10122252; doi:10.1089/vbz.2022.0047)
Supplement: Supplemental data [file Suppl_AppendixTableS2.docx]

**Table 2.** Number of cases (N) and incidence (per 100,000 residents, ± 95% CI) of microbiologically confirmed Lyme borreliosis reported in the National Infectious Diseases Register (NIDR) by year and Finnish hospital districts (HD), 2015–2020.

|  | **2015** | | **2016** | | **2017** | | **2018** | | **2019** | | **2020** | |
| --- | --- | --- | --- | --- | --- | --- | --- | --- | --- | --- | --- | --- |
| **HD** | **N** | **Incidence**  **[95% CI]** | **N** | **Incidence**  **[95% CI]** | **N** | **Incidence**  **[95% CI]** | **N** | **Incidence**  **[95% CI]** | **N** | **Incidence**  **[95% CI]** | **N** | **Incidence**  **[95% CI]** |
| **Ahvenanmaa** | 573 | 1,981.60 [1,827.23; 2,148.73] | 612 | 2,110.56 [1,951.35; 2,282.45] | 658 | 2,250.80 [2,086.94; 2,427.21] | 453 | 1,536.17 [1,401.98; 1,682.99] | 324 | 1,087.65 [976.01; 1,211.90] | 409 | 1,364.52 [1,239.28; 1,502.23] |
| **Etelä-Karjalan** | 57 | 43.26 [33.39;56.04] | 37 | 28.20 [20.46;38.87] | 59 | 45.19 [35.04;58.28] | 62 | 47.74 [37.25;61.19] | 58 | 45.05 [ 34.85;58.23] | 52 | 40.64 [31.00;53.28] |
| **Etelä-Pohjanmaan** | 5 | 2.52 [ 1.08; 5.90] | 3 | 1.58 [ 0.54; 4.65] | 4 | 2.03 [ 0.79; 5.22] | 3 | 1.53 [ 0.52; 4.50] | 6 | 3.09 [ 1.42; 6.74] | 9 | 4.65 [ 2.45; 8.84] |
| **Etelä-Savon** | 11 | 10.59 [ 5.91;18.96] | 4 | 3.87 [ 1.50; 9.95] | 6 | 5.85 [ 2.68;12.76] | 13 | 12.81 [ 7.49;21.92] | 16 | 15.96 [ 9.82;25.93] | 18 | 18.18 [11.50;28.74] |
| **Helsingin ja Uudenmaan** | 582 | 36.39 [33.55;39.47] | 627 | 38.73 [35.82;41.88] | 700 | 42.74 [39.69;46.02] | 729 | 44.14 [41.05;47.46] | 884 | 53.02 [ 49.64;56.63] | 806 | 47.53 [44.36;50.93] |
| **Itä-Savon** | 7 | 15.89 [ 7.70;32.80] | 6 | 13.80 [ 6.32;30.11] | 8 | 18.59 [ 9.42;36.68] | 14 | 33.16 [19.75;55.66] | 7 | 17.05 [ 8.26;35.19] | 10 | 24.78 [13.46;45.61] |
| **Kainuun** | 5 | 6.57 [ 2.81;15.38] | 3 | 3.98 [ 1.35;11.70] | 1 | 1.34 [ 0.24; 7.59] | 1 | 1.35 [ 0.24; 7.65] | 2 | 2.74 [ 0.75; 9.99] | 2 | 2.76 [ 0.76;10.06] |
| **Kanta-Hämeen** | 10 | 5.70 [ 3.10;10.49] | 15 | 8.58 [ 5.20;14.16] | 16 | 9.20 [ 5.66;14.95] | 24 | 13.90 [ 9.34;20.68] | 16 | 9.34 [ 5.75;15.17] | 20 | 11.68 [ 7.56;18.04] |
| **Keski-Pohjanmaan** | 12 | 15.90 [ 9.10;27.79] | 4 | 5.09 [ 1.98;13.09] | 9 | 11.46 [ 6.03;21.78] | 8 | 10.24 [ 5.19;20.21] | 6 | 7.72 [ 3.54;16.84] | 8 | 10.33 [ 5.23;20.38] |
| **Keski-Suomen** | 38 | 15.13 [11.02;20.77] | 34 | 13.50 [ 9.66;18.86] | 35 | 13.85 [ 9.96;19.26] | 36 | 14.23 [10.28;19.70] | 58 | 22.95 [ 17.76;29.66] | 30 | 11.86 [ 8.31;16.93] |
| **Kymenlaakson** | 79 | 45.69 [36.67;56.93] | 76 | 44.22 [35.33;55.34] | 82 | 47.96 [38.64;59.52] | 72 | 42.68 [33.90;53.74] | 112 | 67.22 [ 55.87;80.87] | 71 | 43.02 [34.11;54.25] |
| **Lapin** | 4 | 3.39 [ 1.32; 8.72] | 3 | 2.55 [ 0.87; 7.50] | 7 | 5.94 [ 2.88;12.26] | 4 | 3.41 [ 1.33; 8.77] | 7 | 5.97 [ 2.89;12.32] | 1 | 0.85 [ 0.15; 4.82] |
| **Länsi-Pohjan** | 4 | 6.29 [ 2.45;16.17] | 2 | 3.17 [ 0.87;11.56] | 3 | 4.80 [ 1.63;14.11] | 4 | 6.48 [ 2.52;16.66] | 5 | 8.17 [ 3.49;19.13] | 2 | 3.31 [ 0.91;12.07] |
| **Pirkanmaan** | 17 | 3.24 [ 2.02; 5.19] | 25 | 4.74 [ 3.21; 7.00] | 42 | 7.92 [ 5.86;10.70] | 44 | 8.27 [ 6.16;11.10] | 46 | 8.60 [ 6.45;11.47] | 38 | 7.06 [ 5.14; 9.69] |
| **Pohjois-Karjalan** | 29 | 17.17 [11.96;24.66] | 24 | 14.25 [ 9.58;21.20] | 29 | 17.29 [12.04;24.83] | 34 | 20.43 [14.62;28.55] | 27 | 16.31 [ 11.21;23.73] | 35 | 21.26 [15.29;29.56] |
| **Pohjois-Pohjanmaan** | 16 | 3.92 [ 2.41; 6.37] | 17 | 4.17 [ 2.60; 6.68] | 21 | 5.14 [ 3.36; 7.86] | 21 | 5.13 [ 3.36; 7.84] | 47 | 11.48 [ 8.63;15.26] | 24 | 5.85 [ 3.93; 8.70] |
| **Pohjois-Savon** | 32 | 13.08 [ 9.27;18.46] | 26 | 10.48 [ 7.15;15.36] | 34 | 13.98 [10.00;19.53] | 50 | 20.27 [15.38;26.72] | 53 | 21.58 [ 16.50;28.22] | 45 | 18.39 [13.75;24.60] |
| **Päijät-Hämeen** | 34 | 15.97 [11.43;22.31] | 25 | 12.78 [ 8.66;18.87] | 35 | 16.45 [11.83;22.88] | 26 | 12.27 [ 8.37;17.98] | 29 | 13.73 [ 9.56;19.72] | 19 | 9.03 [ 5.78;14.10] |
| **Satakunnan** | 37 | 16.66 [12.09;22.96] | 29 | 13.16 [ 9.16;18.90] | 60 | 27.46 [21.34;35.34] | 59 | 26.77 [20.76;34.53] | 60 | 27.44 [ 21.32;35.32] | 70 | 32.26 [25.54;40.75] |
| **Vaasan** | 71 | 41.85 [33.18;52.78] | 61 | 35.82 [27.89;46.00] | 65 | 38.20 [29.97;48.68] | 85 | 50.08 [40.51;61.91] | 65 | 38.31 [ 30.06;48.82] | 38 | 22.38 [16.31;30.71] |
| **Varsinais-Suomen** | 290 | 61.20 [54.55;68.66] | 300 | 62.79 [56.08;70.31] | 457 | 95.37 [87.02; 104.52] | 377 | 78.44 [70.91;86.76] | 402 | 83.49 [ 75.72;92.06] | 357 | 73.81 [66.54;81.87] |
| **Total Finland** | 1,913 | 35.01 [33.48;36.61] | 1,933 | 35.38 [33.84;36.99] | 2,331 | 42.37 [40.68;44.12] | 2,119 | 38.44 [36.84;40.11] | 2,230 | 40.41 [ 38.77;42.12] | 2,064 | 37.24 [35.67;38.88] |
